# Global prediction of unreported SARS-CoV2 infection from observed COVID-19 cases

**DOI:** 10.1101/2020.04.29.20083485

**Published:** 2020-05-05

**Authors:** Carson C. Chow, Joshua C. Chang, Richard C. Gerkin, Shashaank Vattikuti

**Affiliations:** 1 Mathematical Biology Section, LBM, NIDDK, National Institutes of Health; 2 Epidemiology and Biostatistics Section, Rehabilitation Medicine, Clinical Center, National Institutes of Health; 3 mederrata; 4 School of Life Sciences, Arizona State University

## Abstract

Estimation of infectiousness and fatality of the SARS-CoV-2 virus in the COVID-19 global pandemic is complicated by ascertainment bias resulting from incomplete and non-representative samples of infected individuals. We developed a strategy for overcoming this bias to obtain more plausible estimates of the true values of key epidemiological variables. We fit mechanistic Bayesian latent-variable SIR models to confirmed COVID-19 cases, deaths, and recoveries, for all regions (countries and US states) independently. Bayesian averaging over models, we find that the raw infection incidence rate underestimates the true rate by a factor, the case ascertainment ratio CAR_t_ that depends upon region and time. At the regional onset of COVID-19, the predicted global median was 13 infections unreported for each case confirmed (CAR_t_ = 0.07 C.I. (0.02, 0.4)). As the infection spread, the median CAR_t_ rose to 9 unreported cases for every one diagnosed as of April 15, 2020 (CAR_t_ = 0.1 C.I. (0.02, 0.5)). We also estimate that the median global initial reproduction number R_0_ is 3.3 (C.I (1.5, 8.3)) and the total infection fatality rate near the onset is 0.17% (C.I. (0.05%, 0.9%)). However the time-dependent reproduction number R_t_ and infection fatality rate as of April 15 were 1.2 (C.I. (0.6, 2.5)) and 0.8% (C.I. (0.2%,4%)), respectively. We find that there is great variability between country- and state-level values. Our estimates are consistent with recent serological estimates of cumulative infections for the state of New York, but inconsistent with claims that very large fractions of the population have already been infected in most other regions. For most regions, our estimates imply a great deal of uncertainty about the current state and trajectory of the epidemic.

## Introduction

Nearly 3 million confirmed cases of the novel coronavirus SARS-CoV-2 infection have been reported worldwide as of late-April, 2020 (“COVID-19 Map” 19). However, it is presumed that many infections remain unreported due to either mildness of symptoms or inadequate testing. Knowledge of the full extent of the COVID-19 pandemic is required to evaluate the effectiveness of mitigation strategies such as social distancing, which have a major economic and social cost.

In the absence of universal testing, a proportion of the infected either do not cross the diagnostic threshold for COVID-19 testing or are unable to acquire medical attention and thus remain undetected. Consequently, the fraction of infected who are reported as cases may be substantially less than 1. Estimating the total case ascertainment ratio (CAR_t_), defined as the ratio of the total number of confirmed cases to the total number of individuals infected with the novel coronavirus SARS-CoV-2 on day t, is thus important for constraining the initial reproduction number R_0_ of COVID-19, as well as recovery and fatality rates due to the disease.

Estimating the true magnitude and dynamics of the fractions of the population who are infected, susceptible, or recovered is a difficult and open problem. Serological and molecular diagnostic tests may not have perfect specificity and sensitivity (a substantial problem if the true positive rates are also low), may not be widely available, and may continue to suffer from ascertainment bias. Recent modeling work has attempted to estimate the true infected population, often relying on reported deaths since there is less ambiguity in the definition (Lourenco et al.; Flaxman et al.). Flaxman et. al 2020 focused on death data from the European Centre of Disease Control and suggest that there are orders of magnitude more infected than detected in confirmed cases. This claim was supported by a separate SIR model fitting cases, case recoveries, and case deaths that predicted a CAR of 1/63 for Italy (Calafiore et al.). Recent, preliminary, serological test results have been mixed. A study from Benavid et. al (Bendavid et al.) is consistent with this high number of undiagnosed cases. However a study of women admitted for delivery estimated that 1 in 8 cases are symptomatic (Sutton et al.), which is consistent with a recent press report by New York state based on sampling grocery store customers (New York Times). Debates continue on the adequacy of these tests.

Here, we develop a set of Bayesian, mechanistic, latent-variable, SIR models (Kermack and McKendrick; Wo and Ag, “Contributions to the Mathematical Theory of Epidemics--II. The Problem of Endemicity.1932”; Wo and Ag, “Contributions to the Mathematical Theory of Epidemics--III. Further Studies of the Problem of Endemicity. 1933”) that respect the uncertainty in the underlying data. We explore multiple specifications and find general consensus among the models. Our models try to account for the effects of mitigation (such as social distancing), the possibility that CAR_t_ (either through changes in definition or in detection, for example via ramp-up of testing) can vary in time, excess variability due to reporting and heterogeneity in the regional population, and incomplete quarantine of reported active cases. A similar model assumed complete quarantine on the basis of debilitating effects of illness or strict adherence to policy (Pedersen and Meneghini), which we relax to quantify the effectiveness of such actions. Using the model, we predict the total number of infected individuals from the confirmed numbers of cases, case recoveries, and case deaths. These three data time-series are sufficient to constrain time-varying estimates for CAR_t_ as well as for the fraction of unobserved infected individuals from which we can obtain the total reproduction number R_t_ and total Infection Fatality Ratio (IFR_t_) as functions of time.

## Methods

Our overall approach uses latent-variable Bayesian models with underlying dynamics given by a family of SIR models (Kermack and McKendrick). We implement our models in the Bayesian inference software language Stan (Carpenter et al.; Hartikainen et al.) to estimate the model statistics within each region independently, using data from The Johns Hopkins University Center for Systems Science and Engineering (JHU CSSE; Dong et al.) and from The COVID Tracking Project (“The COVID Tracking Project”). Collectively, these data consist of daily counts for the reported new cases, deaths, and recoveries (as well as key regional mitigation dates, where available (Flaxman et al.; Mervosh et al.)). We only consider regions that report all three of these quantities, which eliminates several US States and dates beyond April 15 for which the complete data was unavailable.

In the SIR models that we use to quantify the regional population dynamics of COVID-19, interactions between susceptible individuals (*S*) and infected individuals (*I*) induce an increase in *I* and a concomitant decrease in S. The unobserved members of *I* can either transition into a reported SARS-CoV-2 case, *C,* or become no longer infectious (either by recovering or dying). Confirmed cases *C* can then either recover (*R*_*C*_) or die (*D*_*C*_) and also infect more *S* at a rate that can differ from *I*. Mitigation efforts (including any social or policy changes aimed at reducing transmission rates) are modeled by a time dependence on the rate of infectious transmission. This rate changes from an initial value to a final value on a specified day with a 5 day transition period. We also include a similar time dependence in the rate of transition from *I* to *C* to account for changes in testing regimens, clinical classification, and awareness of infected individuals to seek medical attention. We consider six variations of the latent-variable SIR model of varying complexity. Besides the standard nonlinear SIR models, we also fit linear approximations that assume that S is approximately fixed (i.e. the epidemic is small enough that herd immunity is not significantly affecting transmission). The linear models have exponential growth with growth rates that can vary in time including becoming negative.

**Figure 1:**
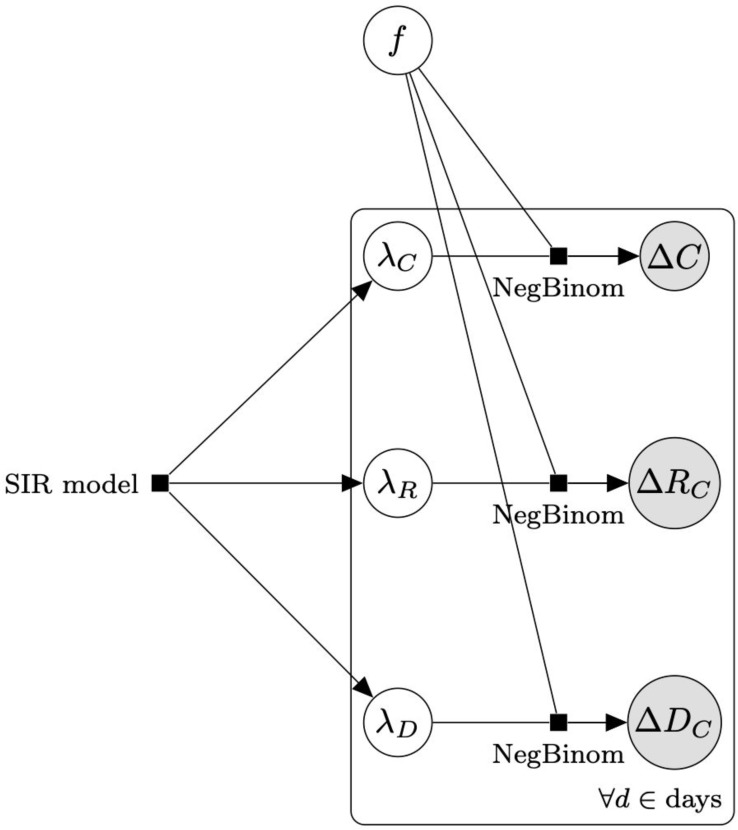
Diagram of generative observation model. The SIR models consist of differential equations that describe the evolution of the S, I, C, R_C_, and D_C_ variables. The models generate the time dependent rate of appearance of cases (λ_*C*_ ), case recoveries (λ_*R*_), and case deaths (λ_*D*_). These rates predict the observable data of daily new cases Δ*C*, new case recoveries Δ*R*_*C*_, and new case deaths Δ*D*_*C*_ with a Negative Binomial likelihood function with an excess variance (*f*λ)^2^, compared to a Poisson likelihood. An expanded version of this diagram with additional details is available in Fig. S1.

We tie each underlying SIR model to the available data using the generative probabilistic scheme shown in Figure 1. We assume a Negative Binomial likelihood, where the variance is a fitted parameter, for the observable data given the model predictions. This formulation accounts for the inherent variability of an underlying stochastic birth-death process (which usually obeys a Poisson distribution), disease progression effects, heterogeneous mixing of population clusters, and errors in classification and reporting inherent in the available data. We modeled prior probabilities for recovery and death rates using inverse gamma distributions derived from ref. (Verity et al.). For the other unknown distributions, we used weakly-informative priors for satisfying positivity and providing numerical and inferential stability. Priors for the day of mitigation application, taken from ref. (Flaxman et al.) and (Mervosh et al.), were used when available. We assessed models by balancing the likelihood of observing the data (given each model) against model complexity using a standard Bayesian model comparison metric such as Leave-One-Out cross validation (LOO) and WAIC (Vehtari et al.), to avoid overfitting. On the basis of this and other Bayesian metrics, we also report model-averaged statistics that take into account model specification uncertainty (Yao et al.).

Computer code written for this project is available at https://github.com/nih-niddk-mbs/covid-sicr.

## Results

**Figure 2:**
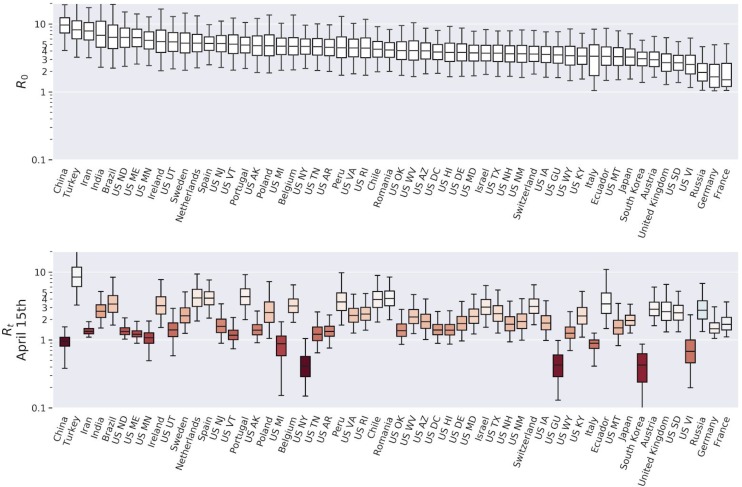
Estimates of the reproduction number *R*_*t*_ across selected regions and time. Upper panel: *R*_*0*_ for selected regions. Bars show median, interquartile range, and 95% credible interval for each region. Lower panel: R_t_ on April 15th in all regions. Color indicates change from R_0_ (more red is more reduction).

**Figure 3:**
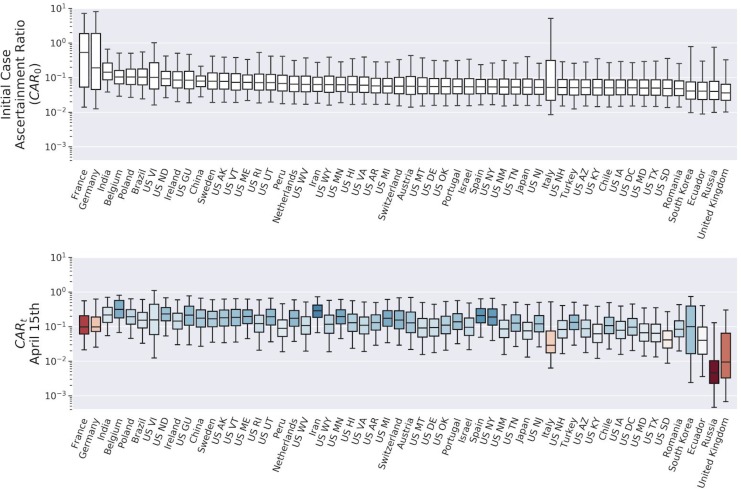
Estimates of the case ascertainment ratio (CAR_t_) across selected regions and time. Upper panel: Initial CAR_t_ for selected regions. Bars show median, interquartile range, and 95% credible interval for each region. Lower panel: CAR_t_ on April 15th in all regions. Color indicates change from initial CAR (more red is more reduction).

**Figure 4:**
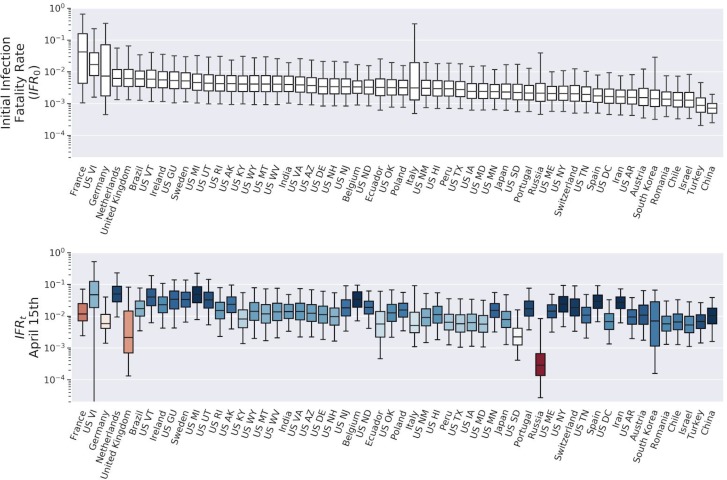
Estimates of the infection fatality rate IFR_t_ across selected regions and time. Upper panel: Initial IFR_t_ for selected regions. Bars show median, interquartile range, and 95% credible interval for each region. Lower panel: IFR_t_ on April 15th in all regions. Color indicates change from initial IFR (more red is more reduction).

Global estimates for R_t_, CAR_t_, and IFR_t_, for 25 representative countries (selected based on case counts) and US states with the prerequisite data, are shown in Figures 2–4 (see Supplementary Material Table S1 for every region analyzed). Overall, we found a consensus between the different model predictions, including between linear and nonlinear models (Supplementary Material Figure S2). Results presented are Bayesian LOO weighted model averages. CAR_t_ can be greater than 1 in rare cases where more cases were introduced by migration at outbreak onset than disappeared from the infected pool without being detected. Credible intervals were broad for most region specific estimates (see Supplementary Material Table S1).

**Figure 5:**
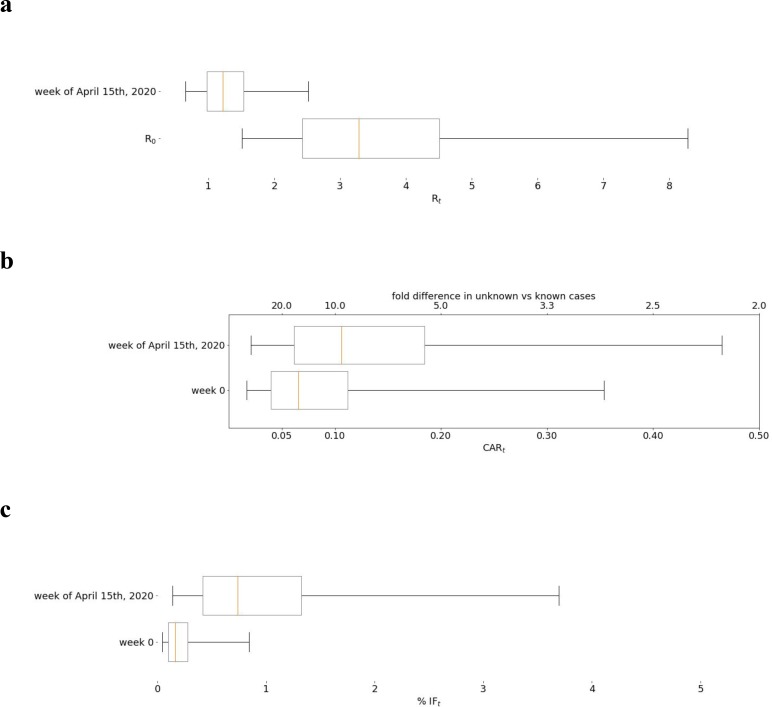
Global distributions of (a) R_t_, (b) CAR_t_, and (c) IFR_t_ using average across models. Figure shows quantiles median, interquartile range, and 95% credible interval.

Global average quantiles across all regions and models are shown in Figures 2–5. The models capture the dynamics of R_t_ (Fig. 2), CAR_t_ (Fig. 3), and IFR_t_ (Fig. 4). The global median R_0_ across all regions was 3.3 (C.I (1.5,8.3), Fig. 5). In the first week of the onset of COVID-19 appearance in a region (which differs by date for each region), we found that for every 1 case identified there are approximately 13 other infections unreported (CAR_t_ (week 0) of 0.07 (C.I (0.02,0.4)). The infection fatality ratio during the first week from regional onset has a global median of 0.0017 (C.I. (0.0005,0.009)) or 0.17%. As the infection spreads these estimates evolve over time such that the R_t_ on the week of April 15th, 2020 was 1.2 (C.I (0.6,2.5)). While R_t_ fell, the global median CAR_t_ and IFR_t_ increased over the same time period to 1 in 10 for CAR_t_ (April 15th, 2020) = 0.1 (C.I. (0.02,0.5)) (a larger fraction of infections ultimately detected) and 0.008 (C.I. (0.002,0.04)) or 0.8% for IFR_t_ (April 15th, 2020). IFR_t_ will be lower than the true mortality rate since it does not account for unreported deaths due to COVID-19. We have also not corrected for the fact that some of the current cases are critically ill and may die in the future. We should also note that since the onset date of COVID-19 varies regionally, the global median as of April 15 is an aggregate over different stages of regional pandemic progression.

**Figure 6:**
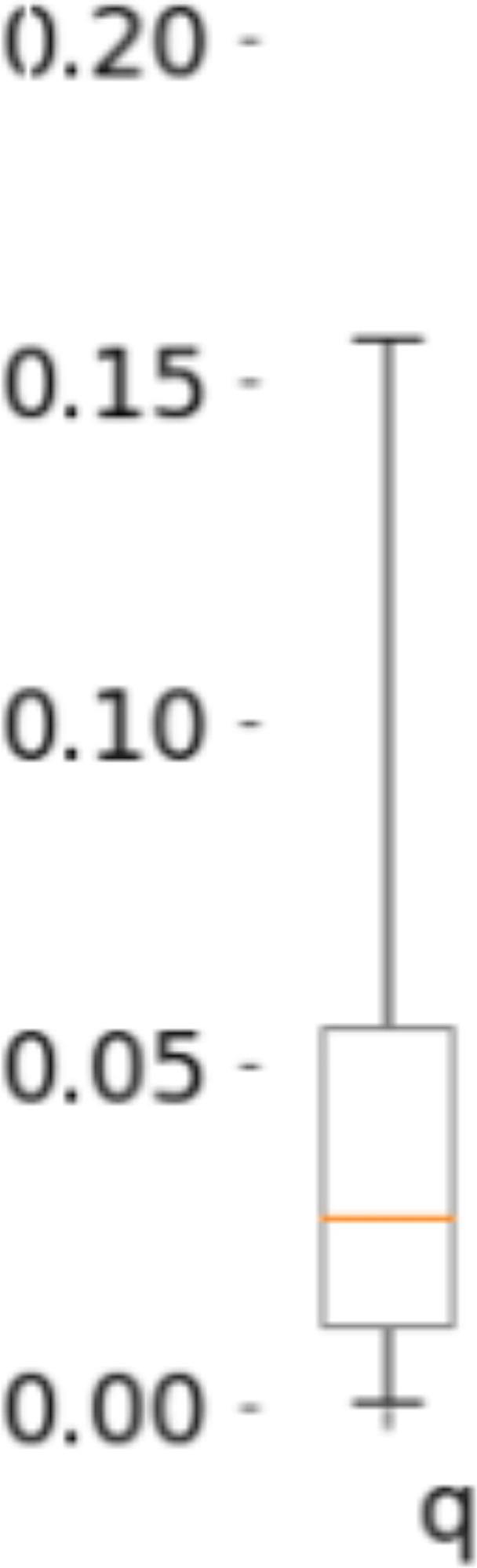
Effectiveness of quarantine. Shows a median q of 0.03 (C.I. (0.001, 0.16)) in the relative infection rate from active known cases. A value of q=0 would correspond to perfect quarantine of active cases (no new infections result from a case once it is identified). See Figure S2 for regional details.

We also evaluated the effects of quarantine and excess variability. Across models we observed a substantial decrease in infection rate by active cases. However, the residual infection rate was still above zero with broad uncertainty. The infectiousness of active cases (the known infected) across all regions was much lower than that of the remaining (unreported) infected (Figure 6), with a median global q = 0.03 (C.I. (0.001,0.16)). While the median global quarantine attenuation was large, there was a broad distribution with some areas having smaller degrees than others. Within Europe, Italy had the smallest median case infection rate consistent with strong quarantine measures (Pedersen and Meneghini) (see Supplementary Figure S3 for regional details).

Variability in reported data likely leads to overdispersion, i.e. to counts that are more variable than would be expected from a Poisson distribution. We thus accounted for and analyzed overdispersion by using a negative binomial distribution in which the excess variance is modeled as (*f*λ)^2^ where λ is the mean rate for that day and *f* is an overdispersion factor that we estimate. As shown in Supplementary Material Figure S4, *f* was approximately 1.5 across all regions.

## Discussion

We use our Bayesian mechanistic models to estimate the extent of COVID-19 prevalence, from publicly-available data. Importantly, we quantify the uncertainty of our estimates, finding them to be large in some regions. Our models perform this estimation task under simplified but plausible mechanisms for infection transmission, case detection, recovery, death, and disease spread mitigation.

The advantage of mechanistic models over non-mechanistic curve extrapolation (Murray and IHME COVID-19 health service utilization forecasting team) is that the model parameters have direct mechanistic interpretations that can be validated independently. More mechanisms and details can also be added incrementally.

### Estimate of the Case Ascertainment Ratio (CAR)

We show that the unaccounted cases exceed the reported by 10 to 20 fold, which is consistent with two independent groups in New York (one using PCR testing) (Sutton et al.; New York Times) but not with a similar test in Santa Clara (Bendavid et al.). However, factors such as false positive rates of the serological tests and selection bias could affect outcomes. Furthermore, the studies cited were not drawn from random samples of the population. Until wide-spread serological testing is implemented, the true proportion of cumulative infections will remain unknown, but we believe that our results provide a credible range for this proportion.

Our model could provide guidance on the effectiveness of various policies for reducing infectiousness until more complete data is obtained. Similar attempts have been made to model these factors. However, they have relied on priors for R_0_ based on confirmed cases (Lourenco et al.; Flaxman et al.; Ferguson et al.), restricted data to reported deaths or cases only (Flaxman et al.; Pedersen and Meneghini; Murray and IHME COVID-19 health service utilization forecasting team), made strong assumptions about quarantine (Pedersen and Meneghini), or ignored the underlying mechanisms that guide the dynamics of epidemics (Murray and IHME COVID-19 health service utilization forecasting team). We are able to improve identifiability of key parameters compared to previous results as in (Lourenco et al.) because we use more information. The motivation to use only case death data is understandable as it may be a more reliable measure than case counts but it is insufficient to constrain the dynamics alone and thus has much less predictive power. However, as shown in the Supplementary Material (Sec. S2), models that includie cases, case recoveries, and case deaths, provide sufficient information for identifiability of undocumented infections although the uncertainty can be large.

### Estimate of the time-dependent reproduction number (R_t_)

Our model predicts the time dependent reproduction number R_t_ for each region, which can give an estimate as to how well mitigation policies are working. Our results are consistent with social distancing, or other policies reducing transmission, as shown by the decrease in R_t_ relative to R at outbreak onset. Our directly inferred region-wide R_0_ estimate of 3.3 is larger than the mean of priors chosen for some other models (Lourenco et al.; Flaxman et al.; Ferguson et al.), but it is consistent with estimates from other work (Cao et al.; Sanche et al.) including several reviewed in (Liu et al.). Our estimate is close to the upper estimates for Italy by Pedersen and Menegheni (Pedersen and Meneghini) of 3.5 when removing quarantine on active cases. Using a similar linear SIR model with complete quarantine lowered their R_0_ estimate to 2.6. Our estimate of R was robust to assumptions on the effectiveness of quarantine (models without this factor gave similar estimates for R_0_ see Supplementary Material Figure S2). Our estimate of parameter q near zero for Italy implies near perfect quarantine. There was more incomplete quarantine in other regions.

### Policy implications

We found a decrease in R_t_ over time with the global estimate currently near 1, though many regions are still well above this as of April 15. However, R_t_ does not need to be below 1 for herd immunity to stop the spread of COVID-19. When the fraction of infected individuals reaches 1–1/R_t_ regionally, then in the absence of reinfection from outside, herd immunity will quench the pandemic locally (Fine et al.). Although there are regional variations, if the current estimate of R_t_ persists then the pandemic will begin to extinguish itself when 17% (C.I. (0%, 60%)) of the susceptible global population has achieved resistance due to either recovery from prior infection or vaccination (0% means we have herd immunity now, under the current mitigation measures). However, if mitigation measures are lifted and R_t_ returns to R,, then that fraction rises to 70% (C.I. (33%, 88%)) to guard against further waves of illness upon reinfection.

The time-varying models give other notable results. On average CAR_t_ has increased, indicating that a larger proportion of infections are being detected. This might be explained by recognition of symptoms increasing during the pandemic coupled with broader availability of testing. Our estimate of IFR_t_ also increased during the spread of the infection. This is probably due to the fact that early in the pandemic those fated to die would have been less likely to be identified as COVID-19 deaths, or more generally from changes in how deaths are reported over time. Because the IFR that we report uses the conventional definition, it is “right censored” -- at any given time point the number of deaths from the current number of infections will continue to grow for some time -- it could reflect an underestimate of the true mortality rate. Because our models explicitly estimate the rate at which both case and non-case infected either die or recover, one can derive a closed form expression for the true mortality rate.

### Assumptions and Limitations

Driven by mechanistic considerations, we make several simplifying modeling assumptions that we believe to be reasonable. Chiefly, we make the standard assumptions of SIR models. For instance, we ignore heterogeneity within populations and the effect of connectivity between populations. We subsume heterogeneity of populations themselves into the model parameters, which are to be interpreted as population-averaged quantities. While mitigation measures have greatly reduced travel, cross-population transmission may still be occurring at low levels. Although our models account for mitigation and for diagnostic changes, our rigid implementation of these factors is unlikely to fully capture the true time course of their effects. Finally, biologically, our model considers an extremely simplified version of disease progression within individuals, ignoring time inhomogeneous mechanisms that may be significant (Bottcher et al.).

Motivated by statistical considerations, we also consider reduced linear approximations to the SIR models, where changes in the size of the susceptible pool are ignored. Empirically, we find these approximations to be more stable for fitting the pandemic in the early stages than the standard nonlinear SIR model. A complication in using the standard nonlinear SIR model lies in the knowledge of the size of the susceptible population. In addition to the fact that it is not known what fraction of the population has innate immunity, the inherent heterogeneity of the spread of disease in the early stages renders the susceptible population estimate problematic if not impossible. (In our nonlinear models we fit it as an adjustable parameter). The linearized model circumvents this difficulty by scaling out the population, and to some degree ameliorates the homogeneous assumption, by averaging over the initial growth of the local clusters. Hence, for the linearized model during the early stages of an epidemic, heterogeneity in infectiousness has less of an impact on the underlying aggregate state variables, and so does not provide a worse fit than a well-mixed population with the same observed data. Only later in the epidemic when the effects of herd immunity are important will the limitations of a linear model with homogeneous mixing be revealed, although the time dependence in the infectiousness we introduce could also be capturing the effects of herd immunity.

Outside of our modeling assumptions, issues of data quality may affect the accuracy of our estimates. Disparate criteria exist across regions for defining cases and case deaths, oftentimes changing within regions themselves as each epidemic progresses. Additionally, data (including death counts) reported by many regions is likely incomplete or unreliable, as evidenced in large spikes of unexplained deaths not officially recognized as Covid-19 deaths (Covid-19 Data - Tracking Covid-19 Excess Deaths across Countries | Graphic Detail | The Economist); while we believe that systematic under-testing can be overcome up to a point, totally inconsistent or fraudulent reporting cannot be.

### Future Extensions

Additional factors need to be explored in future models. For example, a more robust and flexible implementation of mitigation, as well as heterogeneity of disease progression within individuals and in their social interactions, is essential. Rather than fitting regions independently it should be possible to fit a hierarchical global model in which region-specific parameters are partially pooled to achieve additional regularization and to narrow credible intervals. The linear model is robust to uncertainty in the size of the initial susceptible population but if the disease progresses such that we begin to reach population saturation, we must use a nonlinear model that explicitly accounts for population size to make reliable parameter estimates although the time dependence in infectiousness can partially mimic this nonlinear effect. We are optimistic that such a transition will be seamless since we find consistency in model predictions between linear and nonlinear models in the current work.

## Supplementary Material

1

## Definitions

R_t_: Reproduction number at time *t*: the average number of secondary infected individuals due to a single infected individual
CFR_t_: Case ascertainment ratio at time t: the cumulative number of cases reported over cumulative number of infections (observed or not)
IFR_t_: Infection fatality ratio at time t: the cumulative number of known deaths over the cumulative number of infections (observed or not) *This will be lower than the mortality rate, as mortality includes unobserved deaths due to infection*
q: Relative infection rate by known active cases: this is relative to the baseline infection rate of unknown infected cases (including asymptomatic carriers). A value of q = 0 is perfect quarantine of active cases
Over-Dispersion Factor, f: Excess variability in counts beyond a Poisson process, modeled as variance = mean + (f * mean)^2^
Credible interval: An interval spanning the middle 95% of the (posterior) distribution for the estimates, i.e. 2.5th to 97.5th percentile
