# Supplementary material for: Global prediction of unreported SARS-CoV2 infection from observed COVID-19 cases
Source: medRxiv. 2020 May 5:2020.04.29.20083485. Preprint. [Version 1] doi: 10.1101/2020.04.29.20083485 (PMC7239078; doi:10.1101/2020.04.29.20083485)
Supplement: 1 [file NIHPP2020.04.29.20083485-supplement-1.pdf]

# Supplementary Material

## Table S1

| Region | $R_0$ (CI) | Rt(April 15th, 2020) (CI) | CAR (week 0) (CI) | CAR(April 15th, 2020) (CI) | IFR (week 0) (CI) | IFR(April 15th, 2020) (CI) | Number of weeks |
|---|---|---|---|---|---|---|---|
| US_AK | 4.8 (1.9, 12.1) | 1.4 (0.91, 2.67) | 0.08 (0.02, 0.39) | 0.18 (0.03, 0.63) | 0.0042 (0.0009, 0.0234) | 0.0235 (0.0039, 0.0956) | 3 |
| US_AR | 4.5 (2.0, 9.7) | 1.33 (0.76, 2.34) | 0.06 (0.02, 0.28) | 0.13 (0.03, 0.5) | 0.0016 (0.0004, 0.0081) | 0.0095 (0.002, 0.0383) | 4 |
| US_AZ | 4.0 (1.8, 8.6) | 1.86 (1.02, 4.01) | 0.05 (0.02, 0.28) | 0.09 (0.02, 0.42) | 0.0037 (0.0009, 0.0234) | 0.0124 (0.0022, 0.068) | 4 |
| US_DC | 3.9 (1.9, 8.0) | 1.4 (0.89, 2.62) | 0.05 (0.02, 0.27) | 0.1 (0.02, 0.45) | 0.0017 (0.0005, 0.0094) | 0.0067 (0.0013, 0.033) | 4 |
| US_DE | 3.8 (1.7, 8.7) | 1.74 (0.97, 3.7) | 0.05 (0.02, 0.31) | 0.09 (0.02, 0.44) | 0.0034 (0.0008, 0.0211) | 0.0113 (0.0019, 0.061) | 4 |
| US_GU | 3.5 (1.6, 7.7) | 0.43 (0.13, 0.98) | 0.08 (0.02, 0.47) | 0.22 (0.03, 0.77) | 0.0053 (0.001, 0.0318) | 0.0341 (0.0043, 0.1389) | 3 |
| US_HI | 3.8 (1.7, 9.2) | 1.39 (0.87, 2.7) | 0.06 (0.02, 0.35) | 0.13 (0.02, 0.56) | 0.003 (0.0007, 0.018) | 0.0113 (0.0018, 0.0549) | 3 |
| US_IA | 3.6 (1.6, 7.6) | 1.77 (0.98, 3.78) | 0.05 (0.01, 0.29) | 0.08 (0.02, 0.39) | 0.0024 (0.0006, 0.0148) | 0.0063 (0.0011, 0.0342) | 4 |
| US_KY | 3.4 (1.5, 7.3) | 2.25 (1.1, 5.2) | 0.05 (0.01, 0.35) | 0.06 (0.01, 0.39) | 0.0041 (0.001, 0.0308) | 0.0082 (0.0014, 0.0553) | 4 |

medRxiv preprint doi: https://doi.org/10.1101/2020.04.29.20083485.this version posted May 5, 2020. The copyright holder for this preprint (which was not certified by peer review) is the author/funder, who has granted medRxiv a license to display the preprint in perpetuity.
This article is a US Government work. It is not subject to copyright under 17 USC 105 and is also made available for use under a CC0 license.

| | | | | | | | |
|---|---|---|---|---|---|---|---|
| **US_MD** | **3.7 (1.7, 7.8)** | **2.22 (1.23, 4.72)** | **0.05 (0.01, 0.29)** | **0.07 (0.01, 0.35)** | **0.0024 (0.0006, 0.0153)** | **0.0056 (0.0011, 0.0318)** | **4** |
| **US_ME** | **6.3 (2.6, 14.0)** | **1.22 (0.9, 1.89)** | **0.07 (0.02, 0.34)** | **0.2 (0.04, 0.62)** | **0.0021 (0.0006, 0.01)** | **0.0145 (0.0032, 0.0489)** | **3** |
| **US_MI** | **4.7 (2.1, 10.2)** | **0.88 (0.15, 1.85)** | **0.06 (0.02, 0.28)** | **0.17 (0.03, 0.61)** | **0.0046 (0.001, 0.0324)** | **0.0484 (0.0078, 0.2245)** | **5** |
| **US_MN** | **5.7 (2.4, 12.7)** | **1.08 (0.5, 1.9)** | **0.06 (0.02, 0.29)** | **0.19 (0.04, 0.61)** | **0.0024 (0.0006, 0.0117)** | **0.0153 (0.0032, 0.0555)** | **4** |
| **US_MT** | **3.3 (1.5, 7.9)** | **1.51 (0.82, 3.46)** | **0.06 (0.02, 0.37)** | **0.09 (0.02, 0.5)** | **0.0041 (0.0009, 0.0296)** | **0.0118 (0.0017, 0.0727)** | **3** |
| **US_ND** | **6.3 (2.4, 15.0)** | **1.33 (1.03, 2.12)** | **0.09 (0.03, 0.42)** | **0.23 (0.05, 0.68)** | **0.0033 (0.0008, 0.0156)** | **0.019 (0.0041, 0.0619)** | **3** |
| **US_NH** | **3.6 (1.7, 7.9)** | **1.71 (0.94, 3.73)** | **0.05 (0.02, 0.29)** | **0.08 (0.02, 0.41)** | **0.0034 (0.0008, 0.0213)** | **0.0097 (0.0016, 0.0548)** | **4** |
| **US_NJ** | **5.2 (2.3, 11.0)** | **1.59 (0.9, 3.4)** | **0.05 (0.02, 0.26)** | **0.12 (0.03, 0.5)** | **0.0034 (0.0008, 0.0203)** | **0.0184 (0.0034, 0.0904)** | **4** |
| **US_NM** | **3.6 (1.6, 8.2)** | **1.87 (0.99, 4.05)** | **0.05 (0.02, 0.31)** | **0.09 (0.02, 0.43)** | **0.003 (0.0007, 0.0197)** | **0.0091 (0.0015, 0.0518)** | **4** |
| **US_NY** | **4.7 (2.2, 9.6)** | **0.41 (0.15, 1.05)** | **0.05 (0.02, 0.26)** | **0.19 (0.04, 0.66)** | **0.0021 (0.0006, 0.0109)** | **0.0239 (0.0047, 0.0933)** | **5** |
| **US_OK** | **4.1 (1.7, 9.5)** | **1.37 (0.86, 2.82)** | **0.05 (0.02, 0.31)** | **0.11 (0.02, 0.53)** | **0.0032 (0.0008, 0.0195)** | **0.0127 (0.0022, 0.0674)** | **3** |
| **US_RI** | **4.4 (1.8, 11.7)** | **2.41 (1.4, 4.82)** | **0.07 (0.02, 0.53)** | **0.12 (0.02, 0.59)** | **0.0042 (0.001, 0.0308)** | **0.0151 (0.0023, 0.0794)** | **4** |
| **US_SD** | **2.7 (1.4, 5.5)** | **2.51 (1.32, 5.22)** | **0.05 (0.01, 0.36)** | **0.04 (0.01, 0.27)** | **0.0022 (0.0006, 0.0172)** | **0.0022 (0.0004, 0.0158)** | **4** |
| **US_TN** | **4.6 (2.1, 10.0)** | **1.22 (0.65, 2.59)** | **0.05 (0.02, 0.26)** | **0.13 (0.03, 0.51)** | **0.0019 (0.0005, 0.0103)** | **0.0108 (0.0021, 0.0482)** | **4** |
| **US_TX** | **3.7 (1.7, 7.9)** | **2.43 (1.26, 5.44)** | **0.05 (0.01, 0.3)** | **0.06 (0.01, 0.35)** | **0.0028 (0.0007, 0.0179)** | **0.0058 (0.0011, 0.035)** | **4** |
| **US_UT** | **5.5 (2.2, 12.9)** | **1.4 (0.59, 2.92)** | **0.07 (0.02, 0.42)** | **0.19 (0.04, 0.67)** | **0.0044 (0.001, 0.0291)** | **0.0326 (0.0054, 0.1436)** | **4** |
| **US_VA** | **4.4 (1.8, 10.2)** | **2.29 (1.26, 4.73)** | **0.06 (0.02, 0.39)** | **0.11 (0.02, 0.52)** | **0.0039 (0.0009, 0.0258)** | **0.014 (0.0022, 0.074)** | **4** |
| **US_VI** | **2.5 (1.2, 6.2)** | **0.68 (0.2, 2.34)** | **0.1 (0.02, 1.02)** | **0.16 (0.01, 1.1)** | **0.0168 (0.0016, 0.2274)** | **0.0475 (-0.0013, 0.5194)** | **2** |

medRxiv preprint doi: https://doi.org/10.1101/2020.04.29.20083485.this version posted May 5, 2020. The copyright holder for this preprint (which was not certified by peer review) is the author/funder, who has granted medRxiv a license to display the preprint in perpetuity.
This article is a US Government work. It is not subject to copyright under 17 USC 105 and is also made available for use under a CC0 license.

| | | | | | | | |
|---|---|---|---|---|---|---|---|
| **US_VT** | **5.0 (2.1, 12.2)** | **1.17 (0.74, 2.15)** | **0.07 (0.02, 0.38)** | **0.19 (0.04, 0.64)** | **0.0059 (0.0012, 0.0408)** | **0.0401 (0.0062, 0.1887)** | **3** |
| **US_WV** | **4.1 (1.7, 10.4)** | **2.18 (1.24, 4.67)** | **0.06 (0.02, 0.37)** | **0.11 (0.02, 0.51)** | **0.0041 (0.0009, 0.0259)** | **0.0137 (0.0021, 0.0756)** | **3** |
| **US_WY** | **3.4 (1.5, 8.5)** | **1.26 (0.7, 2.61)** | **0.06 (0.02, 0.39)** | **0.12 (0.02, 0.57)** | **0.0041 (0.0009, 0.0275)** | **0.0142 (0.002, 0.0785)** | **3** |
| **Afghanistan** | **3.4 (1.5, 11.2)** | **2.4 (1.2, 6.7)** | **0.07 (0.02, 0.58)** | **0.08 (0.01, 0.55)** | **0.0056 (0.0012, 0.0494)** | **0.0134 (0.0018, 0.1039)** | **4** |
| **Albania** | **4.0 (1.6, 13.4)** | **1.52 (0.8, 6.77)** | **0.14 (0.04, 0.59)** | **0.35 (0.08, 0.81)** | **0.0045 (0.0011, 0.0194)** | **0.0322 (0.0072, 0.0827)** | **4** |
| **Algeria** | **3.6 (1.6, 12.3)** | **2.21 (1.2, 5.5)** | **0.08 (0.02, 0.58)** | **0.17 (0.02, 0.67)** | **0.0063 (0.0014, 0.042)** | **0.0271 (0.0039, 0.1134)** | **5** |
| **Andorra** | **3.4 (1.4, 10.0)** | **2.52 (1.09, 8.3)** | **0.07 (0.02, 0.42)** | **0.11 (0.02, 0.55)** | **0.0043 (0.001, 0.028)** | **0.0176 (0.0029, 0.0946)** | **3** |
| **Antigua and Barbuda** | **3.7 (1.2, 13.2)** | **2.76 (0.76, 11.51)** | **0.19 (0.02, 1.46)** | **0.29 (-0.05, 1.66)** | **0.037 (-0.0191, 0.3924)** | **0.1144 (-0.055, 0.8117)** | **2** |
| **Argentina** | **3.9 (1.8, 8.7)** | **3.56 (1.7, 8.19)** | **0.05 (0.01, 0.28)** | **0.09 (0.02, 0.45)** | **0.0025 (0.0006, 0.0141)** | **0.0097 (0.0019, 0.05)** | **4** |
| **Armenia** | **7.9 (2.8, 20.7)** | **5.72 (1.95, 15.25)** | **0.15 (0.04, 0.53)** | **0.32 (0.08, 0.75)** | **0.0042 (0.0009, 0.0162)** | **0.0288 (0.0069, 0.0766)** | **4** |
| **Austria** | **3.0 (1.6, 6.5)** | **2.83 (1.62, 6.01)** | **0.06 (0.01, 0.43)** | **0.13 (0.02, 0.7)** | **0.0015 (0.0004, 0.0122)** | **0.0108 (0.0018, 0.0636)** | **6** |
| **Azerbaijan** | **2.6 (1.3, 7.5)** | **2.52 (1.32, 5.31)** | **0.05 (0.01, 0.38)** | **0.04 (0.01, 0.31)** | **0.0033 (0.0008, 0.0274)** | **0.0042 (0.0007, 0.0331)** | **5** |
| **Bahamas** | **3.6 (1.2, 12.7)** | **2.72 (0.76, 11.02)** | **0.12 (0.02, 1.1)** | **0.21 (0.01, 1.17)** | **0.0251 (0.003, 0.3134)** | **0.0879 (0.0037, 0.7235)** | **3** |
| **Bahrain** | **3.1 (1.4, 9.2)** | **1.72 (1.18, 3.13)** | **0.06 (0.02, 0.33)** | **0.08 (0.02, 0.37)** | **0.0027 (0.0007, 0.0159)** | **0.003 (0.0004, 0.018)** | **6** |
| **Bangladesh** | **2.7 (1.3, 8.8)** | **2.66 (1.3, 6.98)** | **0.05 (0.01, 0.4)** | **0.03 (0.0, 0.25)** | **0.0045 (0.001, 0.0402)** | **0.0046 (0.0005, 0.0387)** | **4** |
| **Barbados** | **5.5 (1.5, 16.8)** | **4.19 (1.09, 14.03)** | **0.14 (0.02, 0.83)** | **0.27 (0.03, 0.96)** | **0.0193 (0.0028, 0.1623)** | **0.0928 (0.005, 0.483)** | **3** |
| **Belarus** | **2.7 (1.3, 7.7)** | **2.6 (1.34, 5.57)** | **0.05 (0.01, 0.38)** | **0.04 (0.01, 0.27)** | **0.0043 (0.001, 0.0363)** | **0.005 (0.0007, 0.0399)** | **5** |
| **Belgium** | **4.7 (2.1, 13.6)** | **3.17 (1.82, 6.47)** | **0.1 (0.03, 0.51)** | **0.32 (0.07, 0.81)** | **0.0033 (0.0009, 0.0168)** | **0.034 (0.0071, 0.0947)** | **5** |

medRxiv preprint doi: https://doi.org/10.1101/2020.04.29.20083485.this version posted May 5, 2020. The copyright holder for this preprint (which was not certified by peer review) is the author/funder, who has granted medRxiv a license to display the preprint in perpetuity.
This article is a US Government work. It is not subject to copyright under 17 USC 105 and is also made available for use under a CC0 license.

| | | | | | | | |
|---|---|---|---|---|---|---|---|
| **Benin** | **3.5 (1.2, 12.2)** | **2.65 (0.75, 10.98)** | **0.15 (0.02, 1.48)** | **0.23 (-0.05, 1.61)** | **0.0303 (-0.0136, 0.3915)** | **0.0887 (-0.0387, 0.8195)** | **2** |
| **Bolivia** | **4.4 (1.6, 13.6)** | **2.58 (1.23, 8.12)** | **0.09 (0.02, 0.61)** | **0.13 (0.02, 0.64)** | **0.0082 (0.0017, 0.0578)** | **0.0288 (0.004, 0.1522)** | **4** |
| **Bosnia and Herzegovina** | **3.5 (1.7, 8.4)** | **2.65 (1.29, 5.92)** | **0.05 (0.01, 0.31)** | **0.09 (0.02, 0.48)** | **0.0021 (0.0005, 0.0132)** | **0.0101 (0.0019, 0.0543)** | **5** |
| **Brazil** | **6.4 (2.2, 19.4)** | **3.38 (1.65, 8.4)** | **0.1 (0.02, 0.55)** | **0.15 (0.03, 0.61)** | **0.006 (0.0013, 0.0342)** | **0.0174 (0.0035, 0.0772)** | **5** |
| **Brunei** | **4.3 (1.5, 12.5)** | **2.61 (0.79, 6.96)** | **0.14 (0.02, 0.59)** | **0.31 (0.03, 0.88)** | **0.0084 (0.0014, 0.0378)** | **0.0629 (0.0068, 0.2025)** | **4** |
| **Bulgaria** | **5.9 (2.3, 15.5)** | **5.15 (1.97, 13.29)** | **0.08 (0.02, 0.38)** | **0.18 (0.04, 0.61)** | **0.0026 (0.0006, 0.0133)** | **0.0203 (0.0043, 0.0745)** | **4** |
| **Burkina Faso** | **4.9 (1.9, 13.1)** | **4.03 (1.58, 11.53)** | **0.09 (0.02, 0.42)** | **0.17 (0.04, 0.61)** | **0.0043 (0.001, 0.0223)** | **0.0195 (0.0039, 0.0762)** | **3** |
| **Burma** | **2.7 (1.3, 9.4)** | **2.03 (0.88, 8.53)** | **0.06 (0.01, 0.61)** | **0.06 (0.0, 0.61)** | **0.0111 (0.0019, 0.1166)** | **0.0173 (0.0012, 0.2104)** | **2** |
| **Cabo Verde** | **3.4 (1.2, 12.4)** | **2.37 (0.7, 10.1)** | **0.11 (0.02, 1.47)** | **0.15 (0.01, 1.67)** | **0.0221 (-0.0208, 0.3289)** | **0.0525 (-0.0726, 0.733)** | **2** |
| **Cameroon** | **5.7 (1.9, 18.2)** | **4.33 (1.51, 14.69)** | **0.11 (0.02, 0.6)** | **0.21 (0.03, 0.73)** | **0.0086 (0.0014, 0.0519)** | **0.0349 (0.0046, 0.1586)** | **3** |
| **Chile** | **4.2 (2.0, 9.1)** | **3.94 (1.84, 8.94)** | **0.05 (0.01, 0.27)** | **0.11 (0.02, 0.49)** | **0.0013 (0.0003, 0.0073)** | **0.0065 (0.0013, 0.033)** | **5** |
| **China** | **9.6 (4.1, 19.2)** | **0.96 (0.38, 1.56)** | **0.08 (0.03, 0.21)** | **0.16 (0.02, 0.66)** | **0.0007 (0.0002, 0.002)** | **0.0094 (0.0012, 0.0387)** | **11** |
| **Colombia** | **6.6 (2.5, 18.1)** | **4.83 (1.81, 13.04)** | **0.12 (0.03, 0.49)** | **0.25 (0.06, 0.68)** | **0.0031 (0.0007, 0.0137)** | **0.0204 (0.0049, 0.0611)** | **4** |
| **Congo (Brazzaville)** | **3.6 (1.4, 12.5)** | **2.27 (0.7, 9.02)** | **0.09 (0.01, 0.94)** | **0.12 (0.01, 0.96)** | **0.0154 (0.0018, 0.1975)** | **0.036 (0.0016, 0.5313)** | **3** |
| **Congo (Kinshasa)** | **4.1 (1.5, 11.3)** | **2.86 (1.15, 9.25)** | **0.09 (0.02, 0.61)** | **0.15 (0.03, 0.69)** | **0.0103 (0.0022, 0.0732)** | **0.0407 (0.0064, 0.2076)** | **3** |
| **Costa Rica** | **4.7 (1.8, 13.6)** | **3.12 (1.31, 8.73)** | **0.09 (0.02, 0.46)** | **0.17 (0.03, 0.64)** | **0.0037 (0.0008, 0.0215)** | **0.0231 (0.0041, 0.0975)** | **4** |
| **Croatia** | **3.1 (1.5, 7.1)** | **2.65 (1.32, 6.89)** | **0.04 (0.01, 0.28)** | **0.07 (0.01, 0.54)** | **0.0017 (0.0004, 0.0112)** | **0.0058 (0.0006, 0.0498)** | **6** |

medRxiv preprint doi: https://doi.org/10.1101/2020.04.29.20083485.this version posted May 5, 2020. The copyright holder for this preprint (which was not certified by peer review) is the author/funder, who has granted medRxiv a license to display the preprint in perpetuity.
This article is a US Government work. It is not subject to copyright under 17 USC 105 and is also made available for use under a CC0 license.

| | | | | | | | |
|---|---|---|---|---|---|---|---|
| Cuba | 3.7 (1.6, 10.4) | 2.77 (1.28, 8.21) | 0.06 (0.02, 0.45) | 0.08 (0.02, 0.48) | 0.0027 (0.0006, 0.0207) | 0.0074 (0.0013, 0.047) | 4 |
| Cyprus | 6.5 (2.2, 18.0) | 3.73 (1.4, 10.95) | 0.14 (0.03, 0.57) | 0.27 (0.06, 0.74) | 0.0067 (0.0013, 0.0312) | 0.0405 (0.0076, 0.1334) | 4 |
| Czechia | 3.9 (1.8, 8.8) | 2.89 (1.39, 6.27) | 0.06 (0.02, 0.38) | 0.13 (0.03, 0.57) | 0.0016 (0.0004, 0.0108) | 0.0114 (0.0022, 0.0539) | 5 |
| Denmark | 6.0 (2.5, 15.4) | 3.6 (1.53, 8.47) | 0.06 (0.01, 0.34) | 0.18 (0.03, 0.63) | 0.0022 (0.0005, 0.0128) | 0.0194 (0.0035, 0.0765) | 5 |
| Diamond Princess | 3.1 (1.3, 8.3) | 0.29 (0.01, 0.85) | 0.09 (0.02, 0.68) | 0.2 (0.02, 0.88) | 0.0086 (0.0014, 0.0602) | 0.0614 (0.0052, 0.3591) | 9 |
| Djibouti | 4.2 (1.6, 13.7) | 2.54 (0.95, 8.5) | 0.08 (0.02, 0.53) | 0.09 (0.01, 0.57) | 0.0068 (0.0013, 0.049) | 0.012 (0.0014, 0.092) | 2 |
| Dominican Republic | 4.3 (1.9, 10.7) | 4.01 (1.7, 12.67) | 0.05 (0.01, 0.29) | 0.07 (0.01, 0.46) | 0.0031 (0.0006, 0.0204) | 0.0106 (0.0016, 0.0709) | 4 |
| Ecuador | 3.3 (1.5, 8.6) | 3.39 (1.48, 10.94) | 0.04 (0.01, 0.3) | 0.04 (0.0, 0.4) | 0.0032 (0.0006, 0.0253) | 0.0057 (0.0005, 0.0612) | 5 |
| Egypt | 4.9 (1.7, 13.7) | 2.11 (1.13, 6.06) | 0.11 (0.03, 0.62) | 0.18 (0.04, 0.68) | 0.0053 (0.0014, 0.0292) | 0.0213 (0.005, 0.0818) | 4 |
| El Salvador | 5.0 (1.8, 14.2) | 4.14 (1.49, 12.83) | 0.1 (0.02, 0.56) | 0.16 (0.03, 0.65) | 0.0069 (0.0014, 0.0406) | 0.0209 (0.0032, 0.0993) | 2 |
| Estonia | 4.6 (1.9, 12.8) | 3.55 (1.51, 9.52) | 0.07 (0.02, 0.38) | 0.15 (0.03, 0.57) | 0.0031 (0.0007, 0.0174) | 0.0198 (0.0037, 0.0859) | 4 |
| Ethiopia | 2.8 (1.3, 9.6) | 2.12 (0.93, 7.84) | 0.07 (0.01, 0.75) | 0.07 (0.01, 0.72) | 0.0109 (0.0019, 0.1224) | 0.0218 (0.0018, 0.2655) | 3 |
| Finland | 4.8 (1.9, 14.7) | 3.24 (1.53, 7.86) | 0.07 (0.02, 0.46) | 0.15 (0.03, 0.61) | 0.0047 (0.001, 0.0312) | 0.0249 (0.0041, 0.1203) | 5 |
| France | 1.5 (1.1, 5.1) | 1.21 (0.73, 2.55) | 0.53 (0.01, 7.18) | 0.11 (0.02, 0.63) | 0.0422 (0.001, 0.6573) | 0.0163 (0.003, 0.1028) | 10 |
| Gabon | 3.2 (1.3, 10.7) | 2.2 (0.8, 8.6) | 0.07 (0.01, 0.87) | 0.08 (0.01, 0.89) | 0.0129 (0.0019, 0.1629) | 0.023 (0.0016, 0.354) | 2 |
| Georgia | 4.6 (1.7, 12.5) | 1.81 (1.11, 4.45) | 0.09 (0.02, 0.57) | 0.16 (0.03, 0.65) | 0.0056 (0.0013, 0.0364) | 0.0238 (0.0039, 0.1085) | 5 |
| Germany | 1.7 (1.0, 4.9) | 0.9 (0.56, 1.79) | 0.19 (0.01, 8.09) | 0.11 (0.03, 0.71) | 0.0074 (0.0004, 0.3351) | 0.0081 (0.0018, 0.0614) | 10 |

medRxiv preprint doi: https://doi.org/10.1101/2020.04.29.20083485.this version posted May 5, 2020. The copyright holder for this preprint (which was not certified by peer review) is the author/funder, who has granted medRxiv a license to display the preprint in perpetuity.
This article is a US Government work. It is not subject to copyright under 17 USC 105 and is also made available for use under a CC0 license.

| | | | | | | | |
|---|---|---|---|---|---|---|---|
| Ghana | 5.2 (1.7, 15.8) | 3.69 (1.41, 12.56) | 0.1 (0.02, 0.61) | 0.17 (0.03, 0.71) | 0.0105 (0.0019, 0.072) | 0.0361 (0.0039, 0.2002) | 3 |
| Greece | 4.4 (2.0, 12.0) | 2.76 (1.4, 5.82) | 0.06 (0.02, 0.4) | 0.19 (0.03, 0.67) | 0.0038 (0.0008, 0.0247) | 0.0407 (0.0067, 0.1635) | 6 |
| Guatemala | 3.2 (1.3, 10.7) | 2.29 (1.03, 8.5) | 0.07 (0.02, 0.61) | 0.09 (0.01, 0.61) | 0.0091 (0.0018, 0.0768) | 0.0208 (0.002, 0.1621) | 3 |
| Guinea | 4.4 (1.7, 14.3) | 3.51 (1.31, 12.58) | 0.08 (0.02, 0.55) | 0.1 (0.01, 0.6) | 0.0076 (0.0014, 0.0553) | 0.0153 (0.0018, 0.1137) | 2 |
| Guyana | 3.5 (1.2, 12.0) | 2.22 (0.64, 9.12) | 0.16 (0.02, 1.34) | 0.25 (-0.07, 1.49) | 0.0318 (-0.0086, 0.3779) | 0.1119 (-0.0393, 0.8531) | 3 |
| Haiti | 3.6 (1.2, 12.6) | 2.88 (0.81, 11.47) | 0.15 (0.02, 1.18) | 0.24 (0.01, 1.25) | 0.0308 (0.0019, 0.3536) | 0.0929 (-0.0005, 0.7396) | 2 |
| Honduras | 3.9 (1.6, 12.7) | 2.85 (1.24, 8.47) | 0.07 (0.02, 0.48) | 0.13 (0.02, 0.62) | 0.0089 (0.0016, 0.0672) | 0.0384 (0.0052, 0.2132) | 4 |
| Hungary | 5.0 (2.0, 14.0) | 3.23 (1.49, 7.81) | 0.07 (0.02, 0.42) | 0.14 (0.03, 0.55) | 0.0035 (0.0008, 0.0203) | 0.0177 (0.0035, 0.0742) | 5 |
| Iceland | 4.4 (1.8, 14.5) | 2.58 (1.34, 7.08) | 0.11 (0.02, 0.48) | 0.31 (0.06, 0.77) | 0.0026 (0.0006, 0.012) | 0.0098 (0.0017, 0.0419) | 5 |
| India | 6.8 (2.3, 23.6) | 2.64 (1.5, 5.17) | 0.14 (0.04, 0.67) | 0.22 (0.05, 0.7) | 0.004 (0.001, 0.0194) | 0.014 (0.0034, 0.0484) | 5 |
| Indonesia | 5.6 (2.3, 14.5) | 5.21 (2.1, 14.94) | 0.05 (0.01, 0.31) | 0.12 (0.02, 0.6) | 0.0021 (0.0005, 0.0133) | 0.0159 (0.0031, 0.0845) | 5 |
| Iran | 7.9 (3.2, 17.4) | 1.34 (1.1, 1.86) | 0.06 (0.02, 0.28) | 0.29 (0.07, 0.73) | 0.0016 (0.0004, 0.0075) | 0.0272 (0.0062, 0.0722) | 7 |
| Iraq | 2.5 (1.3, 9.6) | 1.65 (1.11, 3.18) | 0.09 (0.02, 0.79) | 0.2 (0.03, 0.8) | 0.0036 (0.0008, 0.0295) | 0.0188 (0.0027, 0.0789) | 6 |
| Ireland | 5.5 (2.0, 16.6) | 3.2 (1.53, 7.74) | 0.08 (0.02, 0.51) | 0.14 (0.03, 0.6) | 0.0055 (0.0012, 0.0354) | 0.0229 (0.0042, 0.1075) | 5 |
| Israel | 3.7 (1.8, 8.3) | 3.05 (1.54, 6.34) | 0.05 (0.02, 0.34) | 0.1 (0.02, 0.48) | 0.0013 (0.0003, 0.0083) | 0.0053 (0.0011, 0.0284) | 5 |
| Italy | 3.4 (1.0, 8.4) | 0.83 (0.21, 1.19) | 0.05 (0.01, 5.16) | 0.03 (0.01, 0.54) | 0.0031 (0.0005, 0.3228) | 0.0062 (0.0014, 0.1103) | 10 |
| Jamaica | 2.2 (1.2, 6.2) | 0.94 (0.5, 3.55) | 0.05 (0.01, 0.46) | 0.04 (0.01, 0.4) | 0.0091 (0.0018, 0.0839) | 0.0121 (0.0012, 0.1249) | 3 |

medRxiv preprint doi: https://doi.org/10.1101/2020.04.29.20083485.this version posted May 5, 2020. The copyright holder for this preprint (which was not certified by peer review) is the author/funder, who has granted medRxiv a license to display the preprint in perpetuity.
This article is a US Government work. It is not subject to copyright under 17 USC 105 and is also made available for use under a CC0 license.

| | | | | | | | |
|---|---|---|---|---|---|---|---|
| Japan | 3.3 (1.5, 7.2) | 1.93 (1.25, 3.36) | 0.05 (0.02, 0.41) | 0.08 (0.01, 0.44) | 0.0023 (0.0006, 0.0168) | 0.0081 (0.0013, 0.0481) | 10 |
| Jordan | 6.1 (2.2, 16.9) | 5.31 (1.91, 15.22) | 0.12 (0.03, 0.48) | 0.28 (0.06, 0.73) | 0.0049 (0.0011, 0.022) | 0.0274 (0.0054, 0.0855) | 3 |
| Kazakhstan | 5.4 (2.0, 14.4) | 3.14 (1.36, 9.36) | 0.1 (0.03, 0.53) | 0.16 (0.04, 0.61) | 0.0034 (0.0009, 0.0177) | 0.0131 (0.0029, 0.0526) | 4 |
| Kenya | 4.2 (1.7, 12.9) | 3.45 (1.41, 10.74) | 0.08 (0.02, 0.5) | 0.13 (0.02, 0.61) | 0.0057 (0.0011, 0.0397) | 0.0203 (0.0028, 0.1114) | 3 |
| Korea, South | 3.1 (1.4, 5.8) | 0.43 (0.05, 0.87) | 0.04 (0.01, 0.79) | 0.1 (0.0, 0.74) | 0.0014 (0.0003, 0.0287) | 0.0071 (0.0002, 0.066) | 9 |
| Kosovo | 2.4 (1.2, 8.0) | 1.88 (0.91, 7.16) | 0.06 (0.02, 0.47) | 0.06 (0.01, 0.5) | 0.0076 (0.0015, 0.0671) | 0.0143 (0.0017, 0.1404) | 2 |
| Kuwait | 4.3 (1.4, 15.9) | 1.79 (1.15, 3.5) | 0.07 (0.02, 1.09) | 0.09 (0.01, 0.73) | 0.0037 (0.0009, 0.0506) | 0.01 (0.0007, 0.0893) | 6 |
| Kyrgyzstan | 5.2 (1.8, 15.2) | 3.71 (1.44, 12.44) | 0.11 (0.02, 0.6) | 0.17 (0.03, 0.68) | 0.0075 (0.0015, 0.0452) | 0.026 (0.0041, 0.1207) | 3 |
| Latvia | 4.7 (1.8, 14.4) | 3.06 (1.26, 9.05) | 0.09 (0.02, 0.53) | 0.19 (0.03, 0.71) | 0.007 (0.0013, 0.0465) | 0.0406 (0.0058, 0.1909) | 4 |
| Lebanon | 4.3 (1.9, 13.3) | 2.53 (1.2, 5.94) | 0.07 (0.02, 0.49) | 0.2 (0.03, 0.71) | 0.0033 (0.0007, 0.0221) | 0.0321 (0.005, 0.1285) | 5 |
| Liberia | 4.9 (1.6, 15.3) | 4.3 (1.27, 14.09) | 0.12 (0.02, 0.8) | 0.18 (0.02, 0.87) | 0.0189 (0.0025, 0.1474) | 0.0427 (0.0032, 0.3093) | 1 |
| Libya | 3.9 (1.3, 13.4) | 3.18 (0.99, 12.24) | 0.1 (0.02, 1.01) | 0.13 (0.01, 1.11) | 0.0178 (0.0012, 0.2248) | 0.0331 (0.0017, 0.4473) | 1 |
| Liechtenstein | 4.6 (1.3, 15.4) | 2.66 (0.62, 10.71) | 0.21 (0.02, 1.31) | 0.38 (-0.05, 1.51) | 0.0332 (-0.0119, 0.3348) | 0.1426 (-0.0647, 0.841) | 3 |
| Lithuania | 5.3 (2.1, 15.5) | 4.22 (1.67, 11.85) | 0.08 (0.02, 0.44) | 0.19 (0.04, 0.66) | 0.0038 (0.0008, 0.0241) | 0.0288 (0.0051, 0.1178) | 4 |
| Luxembourg | 6.1 (2.5, 15.9) | 4.69 (1.85, 12.82) | 0.08 (0.02, 0.43) | 0.3 (0.06, 0.78) | 0.0038 (0.0008, 0.0223) | 0.0535 (0.0096, 0.1865) | 4 |
| Malaysia | 1.8 (1.2, 3.4) | 1.12 (0.72, 1.76) | 0.03 (0.01, 0.17) | 0.2 (0.05, 0.46) | 0.0015 (0.0005, 0.0074) | 0.0111 (0.0027, 0.0305) | 10 |
| Mali | 7.1 (2.2, 19.7) | 5.93 (1.99, 18.52) | 0.15 (0.04, 0.67) | 0.25 (0.06, 0.76) | 0.0124 (0.0027, 0.0606) | 0.0409 (0.0077, 0.1521) | 2 |

medRxiv preprint doi: https://doi.org/10.1101/2020.04.29.20083485.this version posted May 5, 2020. The copyright holder for this preprint (which was not certified by peer review) is the author/funder, who has granted medRxiv a license to display the preprint in perpetuity.
This article is a US Government work. It is not subject to copyright under 17 USC 105 and is also made available for use under a CC0 license.

| | | | | | | | |
|---|---|---|---|---|---|---|---|
| **Malta** | **4.5 (1.7, 12.9)** | **2.43 (1.15, 7.18)** | **0.09 (0.02, 0.6)** | **0.16 (0.03, 0.69)** | **0.0081 (0.0016, 0.056)** | **0.0344 (0.0049, 0.1712)** | **4** |
| **Mauritius** | **5.8 (1.8, 17.2)** | **4.4 (1.46, 14.06)** | **0.13 (0.02, 0.59)** | **0.26 (0.04, 0.77)** | **0.0086 (0.0015, 0.0455)** | **0.0464 (0.007, 0.1733)** | **3** |
| **Mexico** | **3.0 (1.4, 8.4)** | **2.95 (1.4, 6.71)** | **0.05 (0.01, 0.35)** | **0.04 (0.01, 0.3)** | **0.0038 (0.0009, 0.0307)** | **0.0045 (0.0007, 0.0365)** | **5** |
| **Moldova** | **6.1 (2.2, 17.6)** | **3.79 (1.58, 10.86)** | **0.11 (0.03, 0.53)** | **0.17 (0.04, 0.62)** | **0.0034 (0.0008, 0.0179)** | **0.0138 (0.003, 0.0535)** | **4** |
| **Monaco** | **4.4 (1.4, 12.7)** | **3.21 (1.02, 10.28)** | **0.1 (0.02, 0.82)** | **0.19 (0.02, 0.91)** | **0.0155 (0.0027, 0.1382)** | **0.0668 (0.0067, 0.3948)** | **3** |
| **Montenegro** | **6.3 (2.1, 17.9)** | **4.55 (1.59, 14.45)** | **0.14 (0.03, 0.63)** | **0.26 (0.05, 0.77)** | **0.0104 (0.0018, 0.0538)** | **0.0487 (0.0079, 0.1812)** | **3** |
| **Morocco** | **6.3 (2.5, 15.5)** | **5.69 (2.25, 14.53)** | **0.08 (0.02, 0.35)** | **0.21 (0.05, 0.64)** | **0.0024 (0.0006, 0.011)** | **0.022 (0.0052, 0.0715)** | **4** |
| **Netherlands** | **5.2 (2.3, 13.2)** | **4.15 (1.9, 9.34)** | **0.06 (0.02, 0.31)** | **0.17 (0.04, 0.6)** | **0.0062 (0.0013, 0.0558)** | **0.0498 (0.0094, 0.2306)** | **5** |
| **New Zealand** | **3.3 (1.5, 8.3)** | **3.63 (1.55, 12.14)** | **0.03 (0.01, 0.28)** | **0.03 (0.0, 0.58)** | **0.0015 (0.0003, 0.0124)** | **0.002 (0.0001, 0.0403)** | **5** |
| **Niger** | **5.0 (1.7, 15.6)** | **3.81 (1.26, 12.6)** | **0.09 (0.02, 0.51)** | **0.14 (0.02, 0.66)** | **0.0069 (0.0011, 0.0454)** | **0.0216 (0.0025, 0.1255)** | **2** |
| **Nigeria** | **5.4 (2.0, 13.9)** | **4.04 (1.55, 11.73)** | **0.11 (0.03, 0.48)** | **0.18 (0.04, 0.62)** | **0.0046 (0.0011, 0.0221)** | **0.0183 (0.0039, 0.0684)** | **3** |
| **North Macedonia** | **4.6 (1.9, 13.3)** | **3.47 (1.55, 9.41)** | **0.07 (0.02, 0.46)** | **0.12 (0.02, 0.56)** | **0.0039 (0.0008, 0.0255)** | **0.0163 (0.0027, 0.0827)** | **4** |
| **Norway** | **5.2 (2.2, 14.1)** | **3.4 (1.53, 7.46)** | **0.07 (0.02, 0.36)** | **0.21 (0.04, 0.68)** | **0.0051 (0.0009, 0.1817)** | **0.0542 (0.009, 0.4432)** | **5** |
| **Oman** | **2.6 (1.3, 7.9)** | **2.28 (1.29, 4.58)** | **0.05 (0.01, 0.38)** | **0.04 (0.01, 0.3)** | **0.0043 (0.001, 0.0342)** | **0.0054 (0.0008, 0.0463)** | **6** |
| **Pakistan** | **3.5 (1.6, 8.5)** | **3.36 (1.59, 9.15)** | **0.04 (0.01, 0.28)** | **0.07 (0.01, 0.47)** | **0.0019 (0.0004, 0.0121)** | **0.0066 (0.0006, 0.0461)** | **6** |
| **Panama** | **5.4 (2.0, 15.9)** | **3.38 (1.41, 10.05)** | **0.1 (0.02, 0.49)** | **0.17 (0.04, 0.61)** | **0.0049 (0.0011, 0.026)** | **0.0232 (0.0049, 0.0938)** | **4** |
| **Paraguay** | **3.5 (1.4, 10.6)** | **2.3 (1.12, 7.34)** | **0.07 (0.02, 0.55)** | **0.11 (0.02, 0.61)** | **0.0076 (0.0015, 0.0594)** | **0.0268 (0.0034, 0.1633)** | **4** |

medRxiv preprint doi: https://doi.org/10.1101/2020.04.29.20083485.this version posted May 5, 2020. The copyright holder for this preprint (which was not certified by peer review) is the author/funder, who has granted medRxiv a license to display the preprint in perpetuity.
This article is a US Government work. It is not subject to copyright under 17 USC 105 and is also made available for use under a CC0 license.

| | | | | | | | |
|---|---|---|---|---|---|---|---|
| **Peru** | **4.5 (1.8, 12.9)** | **3.63 (1.66, 9.75)** | **0.07 (0.02, 0.41)** | **0.09 (0.02, 0.46)** | **0.0029 (0.0007, 0.0187)** | **0.0065 (0.0013, 0.0355)** | **4** |
| **Philippines** | **5.7 (2.1, 15.8)** | **2.8 (1.09, 8.22)** | **0.17 (0.05, 0.57)** | **0.34 (0.09, 0.75)** | **0.0059 (0.0015, 0.0207)** | **0.0373 (0.0095, 0.0933)** | **4** |
| **Poland** | **4.8 (1.9, 13.5)** | **2.53 (1.05, 7.23)** | **0.1 (0.03, 0.51)** | **0.19 (0.05, 0.64)** | **0.0031 (0.0008, 0.0158)** | **0.0156 (0.0035, 0.0563)** | **4** |
| **Portugal** | **4.9 (2.2, 10.5)** | **4.33 (1.99, 9.17)** | **0.05 (0.02, 0.31)** | **0.14 (0.03, 0.57)** | **0.0022 (0.0006, 0.0129)** | **0.0172 (0.0037, 0.0761)** | **5** |
| **Qatar** | **7.1 (2.4, 19.4)** | **2.29 (1.22, 6.02)** | **0.14 (0.03, 0.57)** | **0.26 (0.06, 0.72)** | **0.0055 (0.0012, 0.0257)** | **0.0302 (0.0056, 0.1028)** | **5** |
| **Romania** | **4.1 (2.0, 8.3)** | **4.09 (1.98, 8.43)** | **0.05 (0.01, 0.25)** | **0.08 (0.02, 0.43)** | **0.0014 (0.0004, 0.0075)** | **0.0057 (0.0013, 0.0301)** | **5** |
| **Russia** | **1.9 (1.1, 4.6)** | **2.74 (1.33, 6.82)** | **0.04 (0.01, 0.76)** | **0.0 (0.0, 0.13)** | **0.0021 (0.0005, 0.0391)** | **0.0003 (0.0, 0.0084)** | **9** |
| **San Marino** | **4.5 (1.6, 12.1)** | **1.38 (0.85, 3.72)** | **0.1 (0.02, 0.61)** | **0.2 (0.04, 0.74)** | **0.0112 (0.0023, 0.0711)** | **0.0633 (0.0105, 0.2618)** | **5** |
| **Saudi Arabia** | **4.9 (2.0, 12.3)** | **4.17 (1.83, 9.86)** | **0.06 (0.02, 0.32)** | **0.12 (0.03, 0.5)** | **0.0015 (0.0004, 0.0084)** | **0.0079 (0.0016, 0.0354)** | **5** |
| **Senegal** | **3.4 (1.7, 7.6)** | **3.25 (1.64, 7.42)** | **0.05 (0.01, 0.28)** | **0.09 (0.02, 0.46)** | **0.0028 (0.0007, 0.0167)** | **0.009 (0.0016, 0.0477)** | **5** |
| **Serbia** | **5.9 (2.1, 17.3)** | **3.13 (1.36, 9.61)** | **0.12 (0.03, 0.61)** | **0.19 (0.04, 0.68)** | **0.0077 (0.0017, 0.0438)** | **0.0295 (0.0058, 0.1247)** | **4** |
| **Singapore** | **3.8 (1.6, 8.7)** | **2.27 (1.17, 4.72)** | **0.06 (0.02, 0.43)** | **0.01 (0.0, 0.32)** | **0.002 (0.0005, 0.0152)** | **0.0005 (0.0, 0.0157)** | **11** |
| **Slovakia** | **7.0 (2.2, 19.2)** | **3.25 (1.33, 10.2)** | **0.15 (0.04, 0.65)** | **0.28 (0.06, 0.78)** | **0.0092 (0.0019, 0.0456)** | **0.0453 (0.0086, 0.1595)** | **4** |
| **Slovenia** | **7.0 (2.6, 19.4)** | **4.02 (1.59, 9.6)** | **0.12 (0.03, 0.5)** | **0.33 (0.08, 0.78)** | **0.0047 (0.0009, 0.0225)** | **0.0592 (0.0128, 0.1663)** | **5** |
| **Somalia** | **3.7 (1.5, 12.2)** | **2.78 (1.06, 10.19)** | **0.06 (0.01, 0.51)** | **0.06 (0.01, 0.55)** | **0.0105 (0.0016, 0.0958)** | **0.0133 (0.0012, 0.1486)** | **1** |
| **South Africa** | **5.8 (2.3, 17.5)** | **5.01 (1.98, 13.49)** | **0.08 (0.02, 0.45)** | **0.19 (0.04, 0.66)** | **0.0044 (0.0009, 0.0273)** | **0.0285 (0.005, 0.1202)** | **4** |
| **Spain** | **5.2 (2.5, 9.8)** | **4.14 (2.1, 7.65)** | **0.05 (0.02, 0.24)** | **0.21 (0.05, 0.64)** | **0.0017 (0.0005, 0.0079)** | **0.0289 (0.0067, 0.0913)** | **6** |

medRxiv preprint doi: https://doi.org/10.1101/2020.04.29.20083485.this version posted May 5, 2020. The copyright holder for this preprint (which was not certified by peer review) is the author/funder, who has granted medRxiv a license to display the preprint in perpetuity.
This article is a US Government work. It is not subject to copyright under 17 USC 105 and is also made available for use under a CC0 license.

| | | | | | | | |
|---|---|---|---|---|---|---|---|
| **Sri Lanka** | **7.0 (2.4, 17.5)** | **3.11 (1.04, 10.37)** | **0.2 (0.05, 0.62)** | **0.4 (0.1, 0.82)** | **0.0103 (0.0026, 0.0355)** | **0.0689 (0.0166, 0.1615)** | **4** |
| **Sudan** | **3.4 (1.2, 11.8)** | **3.2 (1.03, 11.68)** | **0.11 (0.02, 1.05)** | **0.13 (0.01, 1.13)** | **0.022 (0.0018, 0.2961)** | **0.0414 (0.0013, 0.5452)** | **1** |
| **Sweden** | **5.2 (2.1, 14.4)** | **2.27 (1.25, 5.1)** | **0.08 (0.02, 0.42)** | **0.17 (0.04, 0.6)** | **0.0052 (0.0011, 0.0298)** | **0.0334 (0.0065, 0.1377)** | **6** |
| **Switzerland** | **3.6 (1.8, 8.0)** | **3.12 (1.68, 6.5)** | **0.06 (0.02, 0.33)** | **0.16 (0.03, 0.68)** | **0.002 (0.0005, 0.0124)** | **0.0186 (0.0034, 0.0901)** | **6** |
| **Syria** | **3.2 (1.2, 10.9)** | **2.57 (0.77, 10.07)** | **0.15 (0.02, 1.42)** | **0.22 (-0.01, 1.49)** | **0.0312 (-0.0053, 0.3978)** | **0.0896 (-0.0146, 0.8161)** | **2** |
| **Taiwan*** | **2.0 (1.2, 5.1)** | **0.74 (0.18, 2.0)** | **0.05 (0.01, 0.32)** | **0.08 (0.0, 0.48)** | **0.0045 (0.0011, 0.0324)** | **0.0148 (0.0006, 0.0936)** | **10** |
| **Tanzania** | **2.7 (1.3, 8.8)** | **1.48 (0.65, 5.67)** | **0.06 (0.01, 0.57)** | **0.05 (0.0, 0.58)** | **0.011 (0.0019, 0.1148)** | **0.0145 (0.0009, 0.2131)** | **3** |
| **Thailand** | **1.9 (1.2, 3.5)** | **0.81 (0.18, 1.59)** | **0.04 (0.01, 0.16)** | **0.23 (0.04, 0.56)** | **0.0024 (0.0007, 0.0107)** | **0.016 (0.0027, 0.0556)** | **11** |
| **Togo** | **4.1 (1.4, 11.4)** | **2.94 (1.03, 9.48)** | **0.09 (0.02, 0.73)** | **0.17 (0.02, 0.82)** | **0.0113 (0.0023, 0.09)** | **0.0431 (0.0048, 0.2472)** | **3** |
| **Trinidad and Tobago** | **5.6 (1.9, 17.9)** | **4.3 (1.37, 12.93)** | **0.14 (0.02, 0.61)** | **0.32 (0.04, 0.86)** | **0.0156 (0.0022, 0.0917)** | **0.1051 (0.0128, 0.3855)** | **3** |
| **Tunisia** | **4.7 (1.9, 14.2)** | **3.43 (1.45, 9.6)** | **0.08 (0.02, 0.47)** | **0.16 (0.03, 0.64)** | **0.0056 (0.0011, 0.0359)** | **0.033 (0.0053, 0.1501)** | **4** |
| **Turkey** | **8.2 (3.2, 20.1)** | **8.45 (3.27, 25.28)** | **0.05 (0.01, 0.26)** | **0.13 (0.03, 0.51)** | **0.0009 (0.0002, 0.0046)** | **0.0068 (0.0014, 0.0267)** | **3** |
| **Ukraine** | **6.1 (2.4, 16.8)** | **5.79 (2.28, 16.3)** | **0.07 (0.02, 0.37)** | **0.12 (0.03, 0.5)** | **0.0024 (0.0005, 0.0135)** | **0.0097 (0.002, 0.0437)** | **3** |
| **United Arab Emirates** | **1.8 (1.1, 4.1)** | **2.22 (1.23, 5.1)** | **0.04 (0.01, 0.48)** | **0.01 (0.0, 0.18)** | **0.0035 (0.0008, 0.0409)** | **0.0011 (0.0001, 0.0204)** | **10** |
| **United Kingdom** | **2.7 (1.3, 6.3)** | **2.61 (1.31, 6.58)** | **0.04 (0.01, 0.33)** | **0.01 (0.0, 0.3)** | **0.0061 (0.0013, 0.0649)** | **0.0021 (0.0001, 0.0822)** | **9** |
| **Uruguay** | **6.2 (2.1, 18.8)** | **3.44 (1.13, 11.59)** | **0.13 (0.03, 0.54)** | **0.29 (0.05, 0.77)** | **0.0072 (0.0013, 0.0358)** | **0.0388 (0.0063, 0.139)** | **3** |
| **Uzbekistan** | **4.4 (1.6, 13.7)** | **3.07 (1.31, 10.6)** | **0.08 (0.02, 0.62)** | **0.11 (0.02, 0.62)** | **0.005 (0.0012, 0.0368)** | **0.0134 (0.0021, 0.08)** | **3** |
| **Venezuela** | **8.4 (2.7, 23.2)** | **5.69 (1.86, 17.88)** | **0.23 (0.05, 0.68)** | **0.47 (0.11, 0.87)** | **0.0153 (0.0031, 0.0555)** | **0.0804 (0.0171, 0.2025)** | **3** |

medRxiv preprint doi: https://doi.org/10.1101/2020.04.29.20083485.this version posted May 5, 2020. The copyright holder for this preprint (which was not certified by peer review) is the author/funder, who has granted medRxiv a license to display the preprint in perpetuity.
This article is a US Government work. It is not subject to copyright under 17 USC 105 and is also made available for use under a CC0 license.

| | | | | | | | |
|---|---|---|---|---|---|---|---|
| **West Bank and Gaza** | **3.9 (1.4, 12.0)** | **1.6 (0.99, 3.97)** | **0.09 (0.02, 0.68)** | **0.14 (0.01, 0.7)** | **0.0087 (0.0018, 0.0688)** | **0.0312 (0.0027, 0.1843)** | **5** |
| **Zambia** | **4.0 (1.2, 14.4)** | **2.79 (0.7, 11.78)** | **0.16 (0.02, 1.19)** | **0.29 (-0.04, 1.31)** | **0.0295 (-0.001, 0.3355)** | **0.1194 (-0.0155, 0.7793)** | **3** |
| **Zimbabwe** | **3.5 (1.2, 12.0)** | **2.69 (0.79, 10.72)** | **0.17 (0.02, 1.29)** | **0.25 (-0.01, 1.39)** | **0.0345 (-0.0033, 0.3862)** | **0.1018 (-0.0159, 0.7856)** | **2** |

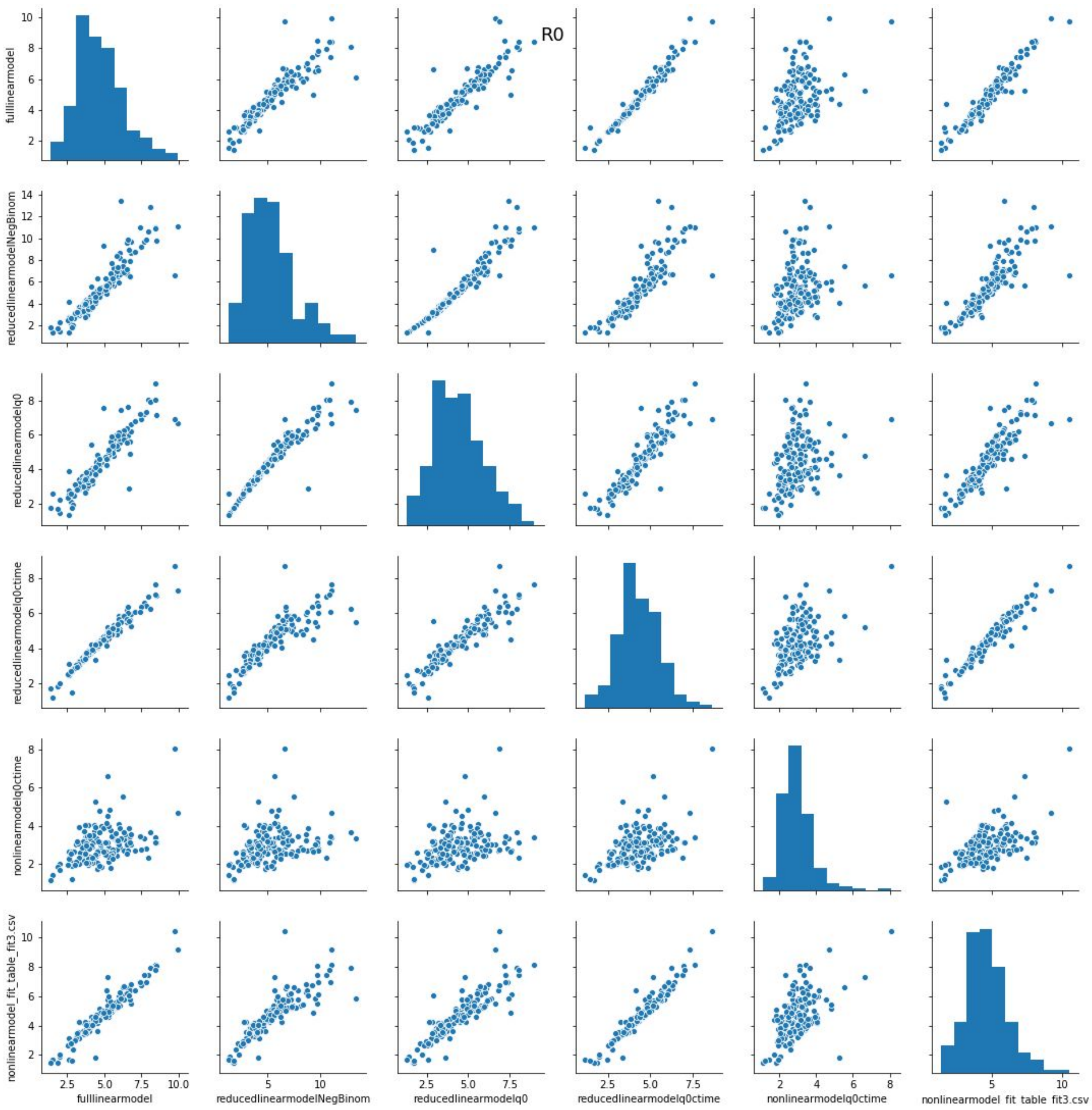


**Figure S2 $R_0$ estimates across models showing model consistency.**

medRxiv preprint doi: https://doi.org/10.1101/2020.04.29.20083485.this version posted May 5, 2020. The copyright holder for this preprint (which was not certified by peer review) is the author/funder, who has granted medRxiv a license to display the preprint in perpetuity.
This article is a US Government work. It is not subject to copyright under 17 USC 105 and is also made available for use under a CC0 license.

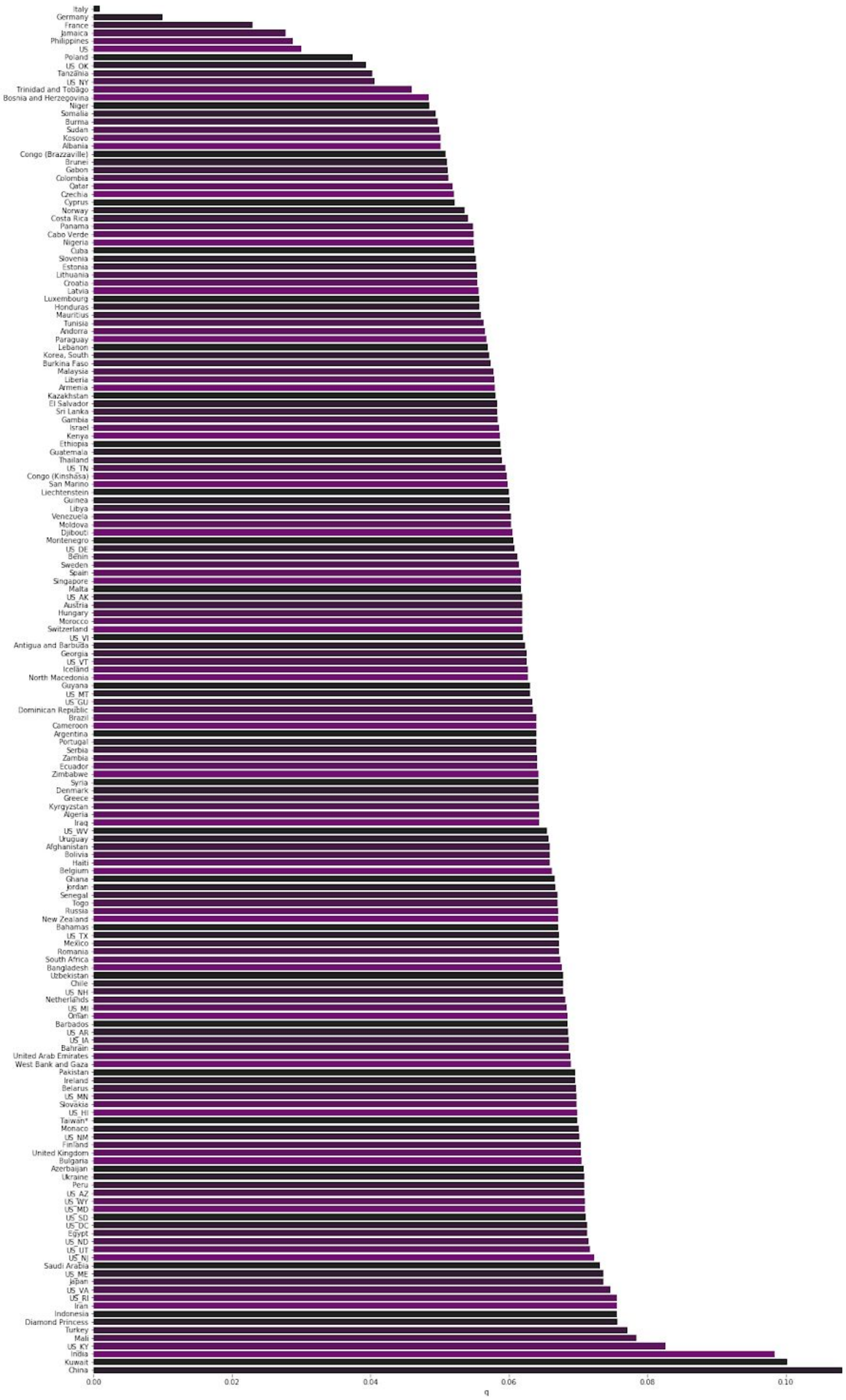

**Figure S3 Median case infectiousness factor q for all regions**

medRxiv preprint doi: https://doi.org/10.1101/2020.04.29.20083485.this version posted May 5, 2020. The copyright holder for this preprint (which was not certified by peer review) is the author/funder, who has granted medRxiv a license to display the preprint in perpetuity.
This article is a US Government work. It is not subject to copyright under 17 USC 105 and is also made available for use under a CC0 license.

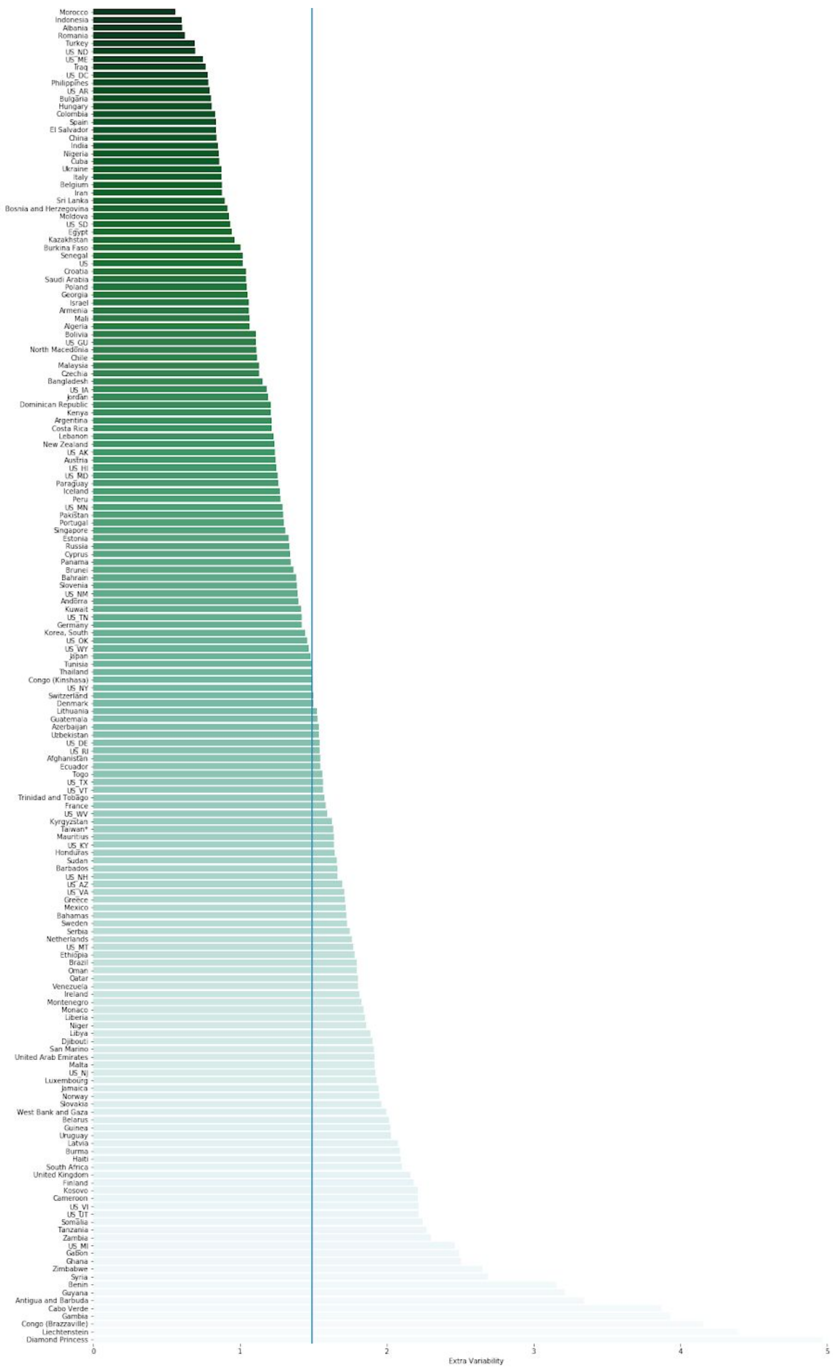


**Figure S4 Median overdispersion factor for all regions.** Blue line is median of 1.5 across all regions.

medRxiv preprint doi: https://doi.org/10.1101/2020.04.29.20083485.this version posted May 5, 2020. The copyright holder for this preprint (which was not certified by peer review) is the author/funder, who has granted medRxiv a license to display the preprint in perpetuity.
This article is a US Government work. It is not subject to copyright under 17 USC 105 and is also made available for use under a CC0 license.

# S1 Latent variable SIR Model

We consider a latent variable SIR model where the observables are cases $C$, case recoveries $R_C$, and case deaths $D_C$. The latent variables are the numbers of susceptible $S$ and undetected infected $I$ individuals. Assuming probability of contact between people is completely random and uniform and ignoring the possibility of births, migration, or deaths due to other causes, the mass action equations for the model are

$$\frac{dS}{dt} = -\frac{\beta}{N}S(I + qC) \tag{S1}$$

$$\frac{dI}{dt} = \frac{\beta}{N}S(I + qC) - \sigma_C I - \sigma_U I \tag{S2}$$

$$\frac{dC}{dt} = \sigma_C I - \sigma_R C - \sigma_D C \tag{S3}$$

where $N$ is the population of the region in question, $\beta$ is the infection rate per contact, $\sigma_C$ is the transition rate from $I$ to $C$, $\sigma_U$ is the disappearance rate of $I$ not transitioning to $C$, and $\sigma = \sigma_R + \sigma_D$ is the disappearance rate for cases. The parameter $q$ accounts for possible differences in virus transmission rate from non-cases. It can be less than one due to say lower social contact or higher due to a larger viral load. If $q = 0$ then the model is identical to an SEIR model.

The equations obey the conservation condition

$$N = S + I + C + R_C + D_C + U$$

where

$$\frac{dR_C}{dt} = \sigma_R C, \tag{S4}$$

$$\frac{dD_C}{dt} = \sigma_D C \tag{S5}$$

$$\frac{dU}{dt} = \sigma_U I. \tag{S6}$$

The population $U$ represents the portion of $I$ that either recover or die but remain undetected.

If initially $S = N$, $I << N$, and the parameters are such that the right hand side of (S2) is positive, then $I$ will grow until it reaches a peak where

medRxiv preprint doi: https://doi.org/10.1101/2020.04.29.20083485.this version posted May 5, 2020. The copyright holder for this preprint (which was not certified by peer review) is the author/funder, who has granted medRxiv a license to display the preprint in perpetuity.
This article is a US Government work. It is not subject to copyright under 17 USC 105 and is also made available for use under a CC0 license.

$\dot{I} = \dot{C} = 0$, which is given by the condition

$$\frac{S^*}{N} = \frac{\sigma(\sigma_C + \sigma_U)}{\beta(\sigma + q\sigma_C)}$$

where $\sigma = \sigma_R + \sigma_D$. The fraction of the population remaining susceptible at the peak of the epidemic is the inverse of the reproduction number

$$R_0 = \frac{\beta(\sigma + q\sigma_C)}{\sigma(\sigma_C + \sigma_U)} \tag{S7}$$

($R_0$ can also be computed using the next generation method). The pandemic will spread if $R_0 > 1$. The total number that becomes infected and thus are no longer susceptible is $N_I = N - S$. The peak of this number is given by

$$N_I^* = N\frac{R_0 - 1}{R_0}, \quad R_0 > 1$$

$I$ will decrease after $N_I$ passes $N_I^*$. Thus, the pandemic can be mitigated by reducing $N_I^*$, which can be achieved by either decreasing $R_0$ or decreasing $N$. We model the effects of mitigation with a time dependence in $\beta$ via $\beta \to \beta_t$ where

$$\beta_t = \beta(m_\infty + \frac{1 - m_\infty}{1 + \exp((t - m_{50})/5)}) \tag{S8}$$

where $t = 0$ is the day of the first case. $\beta_t$ transitions from its initial value to a new value $\beta m_\infty$ on day $m_{50}$ with a transition time of 5 days. We also account for the possibility that the case detection rate can change in time with with $\sigma_C \to \sigma_t$ where

$$\sigma_{Ct} = \sigma_C(c_\infty + \frac{1 - c_\infty}{1 + \exp((t - c_{50})/5)}) \tag{S9}$$

For example, the method of classification of cases changed in China on a single day leading to spike in the number of cases and this time dependence accounts for situations such as that.

The mean field SIR model assumes a well mixed population where the probability of interactions are homogeneous that does not reflect actual human interactions. An actual epidemic, rather than growing homogeneously throughout the population, will be seeded locally and spread within clusters

medRxiv preprint doi: https://doi.org/10.1101/2020.04.29.20083485.this version posted May 5, 2020. The copyright holder for this preprint (which was not certified by peer review) is the author/funder, who has granted medRxiv a license to display the preprint in perpetuity.
This article is a US Government work. It is not subject to copyright under 17 USC 105 and is also made available for use under a CC0 license.

that then propagate to other local clusters. Each cluster may locally saturate before spreading to another cluster and thus the average dynamics of the epidemic over a large region is not reflected by the actual spread at the local level. Hence, the population size $N$ in the SIR model is not simply the regional population but a complex aggregation over many interacting local clusters, which may be difficult to estimate. This is a major limitation of mean field models since $N$ is a major factor in determining the eventual rise and fall of the pandemic. However, this limitation can be circumvented by noting that in the initial stages of the epidemic where $I$ is small and less than even the cluster population, we can fix $S = N$ and the system (S1), (S2), and (S3) reduces to the two dimensional linear system

$$\frac{dI}{dt} = \beta_t I - \sigma_{Ct} I - \sigma_U I + q\beta C \tag{S10}$$

$$\frac{dC}{dt} = \sigma_{Ct} I - \sigma_R C - \sigma_D C \tag{S11}$$

$N$ is scaled out of the dynamics.

The eigenvalues for the linear system (S10) and (S11) assuming that $\beta_t$ and $\sigma_{Ct}$ are constant in time are

$$r_{\pm} = \frac{Tr^2}{2} \pm \frac{1}{2}\sqrt{Tr^2 - 4Det}$$

where $Tr = \beta_t - \sigma_{Ct} - \sigma_U - \sigma$ and $Det = -\sigma(\beta_t - \sigma_{Ct} - \sigma_U) - q\beta\sigma_{Ct}$. For $q = 0$ the eigenvalues simplify to

$$r_+ = \beta_t - \sigma_{Ct} - \sigma_U$$
$$r_- = -\sigma$$

For most parameter values $r_+$ will dominate the temporal dynamics. The pandemic will grow when it is positive and begin to extinguish only when $\beta_t$ falls below $\sigma_{Ct} + \sigma_U$.

We can use the eigenvalues for a lower bound on the rate for the pandemic to extinguish. In the nonlinear equations, we can consider an effective infectiousness $\beta^* = \beta_t S(t)/N = \beta_t(1 - n_I)$, where $n_I$ is the fraction of the population that is immune, either innately or by recovering from the infection. From (S7) we can set

$$\beta_t = R_t(\sigma_{Ct} + \sigma_U)$$

medRxiv preprint doi: https://doi.org/10.1101/2020.04.29.20083485.this version posted May 5, 2020. The copyright holder for this preprint (which was not certified by peer review) is the author/funder, who has granted medRxiv a license to display the preprint in perpetuity.
This article is a US Government work. It is not subject to copyright under 17 USC 105 and is also made available for use under a CC0 license.

for $q = 0$ to obtain

$$r_+ \approx (R_t(1 - n_I) - 1)(\sigma_U + \sigma_C)$$

Thus the pandemic will extinguish at the rate of infection disappearance moderated by the residual spread.

## S2 Parameter identifiability

Here, we investigate whether the parameters in ($S10$) and ($S11$) are in principle identifiable from the observable data. We first consider the case without mitigation in which there are six parameters, $\beta$, $\sigma_C$, $\sigma_R$, $\sigma_D$, $\sigma_U$, and $q$ together with the dynamic variables $I(t)$ and $C(t)$. The observable data consists of the time series of cases, case recoveries, and case deaths, which we denote by $\lambda_C(t)$, $\lambda_R(t)$,and $\lambda_D(t)$, respectively. For this analysis, we assume perfect information with no uncertainty.

From the data, we can immediately construct the following conditions

$$\sigma_C I(t) = \lambda_C(t) \tag{S12}$$
$$\sigma_R C(t) = \lambda_R(t) \tag{S13}$$
$$\sigma_D C(t) = \lambda_D(t) \tag{S14}$$
$$\frac{dC}{dt} = \lambda_C(t) - \lambda_R(t) - \lambda_D(t) \tag{S15}$$

From these conditions we immediately obtain $C(t)$, $\sigma_R$, and $\sigma_D$. Dividing (S10) by (S11), we obtain

$$\frac{dI}{dC} = \frac{\alpha_1 I + q\beta C}{\sigma_C I - \sigma_R C - \sigma_D C} \tag{S16}$$

where $\alpha_1 = \beta - \sigma_U - \sigma_C$. Given that the epidemic is initiated with $I > 0$ and $C = 0$, then prior to the first case arising we can assume $dI/dC = \alpha_1/\sigma_C$, from.which we can integrate to obtain $\sigma_C I(0) = \alpha_1 C(0)$, where $t = 0$ marks the day of appearance of the first case. This then gives $\alpha_1 = \lambda_C(0)/C(0)$.

Multiplying ($S10$) by $\sigma_C$ gives

$$L(t) \equiv d\lambda_C/dt - \alpha_1 \lambda_C = \sigma_C q\beta C \tag{S17}$$

medRxiv preprint doi: https://doi.org/10.1101/2020.04.29.20083485.this version posted May 5, 2020. The copyright holder for this preprint (which was not certified by peer review) is the author/funder, who has granted medRxiv a license to display the preprint in perpetuity.
This article is a US Government work. It is not subject to copyright under 17 USC 105 and is also made available for use under a CC0 license.

and thus $\sigma_C q\beta = L/C$. Finally, we can also rewrite (S10) as

$$\frac{d\lambda_C}{dt} - (\alpha_1 - \sigma_C)\lambda_C = \sigma_C\lambda_C + L(t) \tag{S18}$$

from which $\sigma_C$ can be inferred. Given $\sigma_C$ we can then infer $I(t)$ from $\lambda_C(t)$ from which we can derive conditions for $\beta - \sigma_U$ and $q\beta$, which are only two conditions for three unknowns. Thus, the observable data can at most specify five out of the six unknown parameters.

We can resolve this nonidentifiability in two ways. The first is by specifying one of the parameters. For example, we can specify $\sigma_U$ given that we have a prior on the rate of recovery or death from the infection. We can also specify $q$ since it is a measure of how effective the isolation of cases are. The second is to utilize the fact that mitigation acts as a time dependent perturbation on $\beta$ assuming all the other parameters are fixed. The mitigation is set by two parameters, the day it is applied and the effectiveness in diminishing $\beta$. Essentially the model can be applied separately before and after mitigation where only $\beta$ has a different value. If the day of mitigation is known then this results in four conditions for four unknowns, i.e. $\beta_{before}$, $\beta_{after}$, $q$, and $\sigma_U$ and the parameters can be identified. Thus in principle, with perfect information, the model can be identified if given some prior information.

# S3 Parameter estimation

We estimate the parameters and their uncertainties using Bayesian methods, by putting prior distributions on each of the model parameters. A schematic of our overall Bayesian latent-variable model is shown in Fig. S1

We consider various versions of the model with varying number of parameters and weigh them using model comparison measures such as WAIC and LOO.

Considering the mean field SIR equations as a birth and death process implies a Poisson likelihood. However, in addition to this inherent stochasticity, errors will be introduced due to variability in criteria for measurement and recording. We account for this additional variance with a Negative Binomial

medRxiv preprint doi: https://doi.org/10.1101/2020.04.29.20083485.this version posted May 5, 2020. The copyright holder for this preprint (which was not certified by peer review) is the author/funder, who has granted medRxiv a license to display the preprint in perpetuity.
This article is a US Government work. It is not subject to copyright under 17 USC 105 and is also made available for use under a CC0 license.

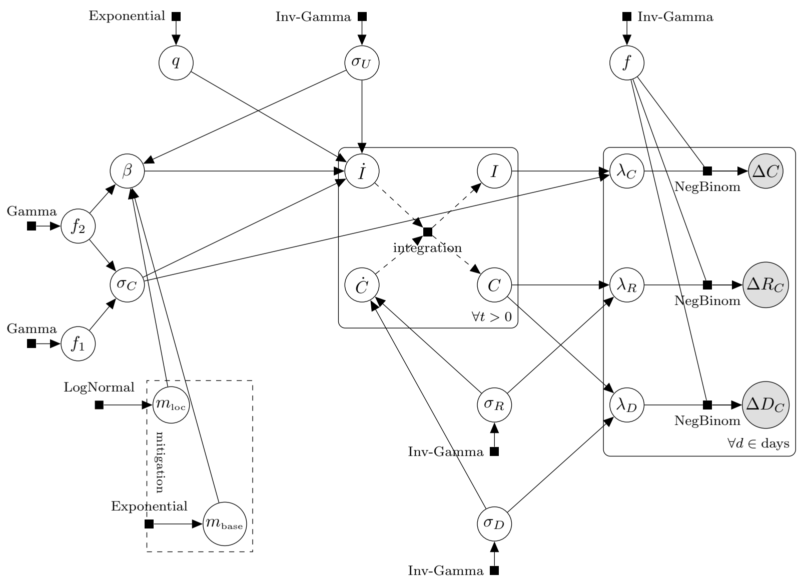


Figure S1: Bayesian Model diagram showing variable dependencies and prior distribution families.

distribution cf.

$$n_C(d) \sim \text{NegBinom}(\sigma_c c(d) I(d), \phi) \tag{S19}$$
$$n_R(d) \sim \text{NegBinom}(\sigma_R C(d), \phi) \tag{S20}$$
$$n_D(d) \sim \text{NegBinom}(\sigma_D C(d), \phi) \tag{S21}$$

where $n_{C,R,D}$ are the total number of new cases, recovered cases, and case deaths on day $d$, $I(d), C(d)$ are the total number for day $d$, and $\phi$ is a fitted factor quantifying the extra variance where $x \sim \text{NegBinom}(\mu, \phi)$ with $E[X] = \mu$ and $\text{var[X]} = \mu + \mu^2/\phi$. We use inverse gamma distributed priors on $\sigma_U$, $\sigma_R$ and $\sigma_D$ using parameters derived from ref X. We fit from the first day in which the daily case count is greater than one, which we call day 0. We then set $C(0)$ to this count and $I(0) = (\beta - \sigma_U - \sigma_C)/\sigma_C C(0)$.

The cumulative population that becomes infected and cumulative cases

medRxiv preprint doi: https://doi.org/10.1101/2020.04.29.20083485.this version posted May 5, 2020. The copyright holder for this preprint (which was not certified by peer review) is the author/funder, who has granted medRxiv a license to display the preprint in perpetuity.
This article is a US Government work. It is not subject to copyright under 17 USC 105 and is also made available for use under a CC0 license.

at time $t$ are given by

$$N_I(t) = \int_0^t \beta m(s)(1 + qC(s))I(s)ds \tag{S22}$$

$$N_C(t) = \int_0^t \sigma_C I(s)ds \tag{S23}$$

The cumulative totals for recovered and dead are $R_C$ and $D_C$. From these quantities we can estimate the case ascertainment ratio, $N_C/N_I$ and the total infection fatality ratio, $D_C/N_I$.

# S4 Data and Software

All data and code can be found at https://github.com/nih-niddk-mbs/covid-sicr. This repository contains several python scripts and Jupyter notebooks for replicating our findings, as described in the README file there. These can be used to obtain new post-publication estimates with additional data, provided that the data providers listed in the main text continue to provide a consistent API. We used Stan, which provides Bayesian inference using a heavily-optimized No-U-Turn sampler, a variant of Hamiltonian Monte Carlo, and each model described here is available as a .stan file. Python 3 was used for all other reported results, including interaction with Stan (via pystan). A subset of the work implemented in Julia is also available in the repository.
